# Diagnostic and prognostic value of m5C regulatory genes in hepatocellular carcinoma

**DOI:** 10.3389/fgene.2022.972043

**Published:** 2022-08-29

**Authors:** Xiawei Yang, Feng Yang, Liugen Lan, Ning Wen, Haibin Li, Xuyong Sun

**Affiliations:** ^1^ Graduate School, Guangxi Medical University, Nanning, China; ^2^ Department of Gynocology, The Second Affiliated Hospital of Guangxi Medical University, Nanning, China; ^3^ Transplant Medical Center, The Second Affiliated Hospital of Guangxi Medical University, Nanning, China; ^4^ Guangxi Key Laboratory of Organ Donation and Transplantation, Nanning, China; ^5^ Guangxi Key Laboratory for Transplantation Medicine, Nanning, China; ^6^ Guangxi Transplantation Medicine Research Center of Engineering Technology, Nanning, China

**Keywords:** hepatocellular carcinoma, HCC, 5-methylcytosine, m5C, biomarkers, prognosis

## Abstract

**Background:** A high mortality rate makes hepatocellular carcinoma (HCC) one of the most common types of cancer globally. 5-methylcytosine (m5C) is an epigenetic modification that contributes to the prognosis of several cancers, but its relevance to HCC remains unknown. We sought to determine if the m5C-related regulators had any diagnostic or prognostic value in HCC.

**Methods:** M5C regulatory genes were screened and compared between HCC and normal tissue from The Cancer Genome Atlas (TCGA)and Gene Expression Omnibus (GEO) databases. Least absolute shrinkage and selection operator method (LASSO) and univariate Cox regression analysis of differentially expressed genes were then performed to identify diagnostic markers. A LASSO prognostic model was constructed using M5C regulatory genes with prognostic values screened by TCGA expression data. HCC patients were stratified based on risk score, then clinical characteristics analysis and immune correlation analysis were performed for each subgroup, and the molecular functions of different subgroups were analyzed using both Gene Set Enrichment Analysis (GSEA) and Gene Set Variation Analysis (GSVA). The prognostic model was evaluated using univariate and multivariate Cox analyses as well as a nomogram. Molecular typing was performed according to m5C regulatory genes and immune checkpoint genes expression respectively, and clinical characterization and immune correlation analysis were performed for each subgroup.

**Results:** M5C regulatory genes are expressed differently in HCC patients with different clinical and pathological characteristics, and mutations in these genes are frequent. Based on five m5C regulators (NOP2, NSUN2, TET1, YBX1, and DNMT3B), we constructed a prognostic model with high predictive ability. The risk score was found to be an independent prognostic indicator. Additionally, risk scores can also be applied in subgroups with different clinical characteristics as prognostic indicators.

**Conclusion:** The study combined data from TCGA and GEO for the first time to reveal the genetic and prognostic significance of m5C-related regulators in HCC, which provides new directions for identifying predictive biomarkers and developing molecularly targeted therapies for HCC.

## Introduction

Hepatocellular carcinoma (HCC) ranks sixth in the cancer incidence worldwide and ranks third in cancer-related deaths (de Martel et al., 2020), and it is a major public health issue. Despite significant advancements in therapy, the 5-year survival rate for advanced HCC is still dismal due to the cancer’s late detection, susceptibility to metastasis, and high recurrence rate. Although some biomarkers, including alpha-fetoprotein (AFP) and heat shock protein 90 (Hsp90), have proven to be useful, the search for early diagnosis biomarkers and effective therapies for HCC patients is urgent.

There is growing evidence that post-transcriptional modifications of RNA are important in different cancers (Cheng et al., 2018; Barbieri and Kouzarides, 2020; Begik et al., 2020; Chu et al., 2022), which provides ideas for developing new treatment modalities. There have been 170 types of modifications identified thus far (Boccaletto et al., 2018), such as N6-methyladenosine (m6A), 5-methylcytosine (m5C) (Wang et al., 2013), 7-methylguanosine, and pseudouridylation (Roundtree et al., 2017; Shi et al., 2020). However, their functions remain widely unknown due to technical limitations in accurate localization throughout the genome (Cohn, 1960; Bauer et al., 2016). There are many post-transcriptional modifications, but the most common is a reversible modification called m5C, which serves different functions in different RNA types (Chow et al., 2007; Squires et al., 2012; Huang et al., 2019; Trixl and Lusser, 2019; He et al., 2020a; Cui et al., 2020). M5C modification involves adenosine methyltransferases (“writers”), demethylases (“erasers”), and “readers” for protein recognition and binding. The “writers” include NSUN1-NSUN7, DNMT1, DNMT2, DNMT3a, and DNMT3b, “erasers” include TET1, TET2, TET3, and ALKBH1, and among the “readers” are ALYREF and YBX1. Abnormal modification of m5C has been connected to many abnormal states, for example mitochondrial dysfunction, abnormal embryogenesis and neurodevelopment, tumorigenesis, and tumor cell proliferation and migration (Navarro et al., 2021; Walworth et al., 2021). It has also been suggested that m5C modification can even alter the fate of cancer cells (Yang et al., 2020), and can be utilized as a biomarker for the prognosis of many kinds of cancers (Gama-Sosa et al., 1983; Chellamuthu and Gray, 2020). One study comprehensively explored and systematically profiled the expression features of m5C-related regulators in HCC and proved the m5C modification patterns play a crucial role in the tumor immune microenvironment and prognosis of HCC (Liu et al., 2022b). In spite of the fact that anomalous RNA m5C modification has been detailed to play numerous capacities in HCC (He et al., 2020b; Sun et al., 2020), the relationship between m5C regulatory genes and HCC is still poorly understood, and the diagnostic and prognostic value of m5C regulatory genes for HCC is unknown.

This study screened and compared the expression characteristics of the m5C regulators in HCC samples with those in normal samples using the expression matrix from TCGA and GEO databases. Univariate Cox as well as LASSO regression analyses were employed to discover diagnostic markers. Then five m5C regulatory genes with prognostic value were screened by using the data from TCGA to construct a prognostic model. To find out if m5C regulatory genes are valuable for diagnosis and prognosis in HCC, researchers performed molecular typing based on m5C regulatory gene and immune checkpoint gene expression, and immune correlate analyses and clinical characteristic analyses were also performed for each subgroup.

## Materials and methods

### Acquired data and identified differentially expressed genes

We obtained Gene expression data from TCGA database (Hutter and Zenklusen, 2018) (https://portal.gdc.cancer.gov/) and the GSE76427 dataset (Grinchuk et al., 2018) (https://www.ncbi.nlm.nih.gov/geo/query/acc.cgi?acc=GSE76427) in the GEO database (Barrett et al., 2007) (https://www.ncbi.nlm.nih.gov/geo/). The TCGA database contains expression data (Table 1), copy number variants (CNVs), single nucleotide polymorphisms (SNPs), and relevant clinicopathological features for 374 HCC samples and 50 paraneoplastic samples. The microarray platform for GSE76427 (sample size: disease group 115/control group 52) (Table 1) dataset is Illumina HumanHT-12 V4.0 expression beadchip, and gene set related to m5C regulators was obtained by Cui et al.'s study (Cui et al., 2021; Wang et al., 2021; Liu et al., 2022a). We first used “sva” package (Leek et al., 2012) to preprocess the downloaded TCGA and GEO dataset expression matrices, including: data background adjustment and normalization, and output the expression of intersecting genes in the two datasets separately. The Perl language was then applied to extract the expression of m5C regulator genes in both datasets. To determine the validity of the grouping, we did a principal component analysis (PCA) and visualized with the help of “ggplot2” package. Subsequently, by using “limma” package, we determined DEGs between HCC and normal liver tissue at *p* < 0.05.

**TABLE 1:** Baseline data.

Data	Normal	Tumor
TCGA	50 (11.8%)	374 (88.2%)
GSE76427	52 (31.1%)	115 (68.9)

### Copy number variant and single nucleotide polymorphism analyses

GISTIC 2.0 was used to find genes with significant amplifications or deletions (Mermel et al., 2011) with thresholds of *p* > 0.1 and *p* < 0.05. Mutsig2 was used to search genes with significant mutations using a threshold of *p* < 0.05.

### Predictive model construction and validation

We used m5C regulator genes to construct a prediction model. The “survival” R package helped us separate HCC patients into high- and low-risk groups, then we identified significant RNA regulator genes through univariate Cox analysis, and visualized through R package “forestplot.” The R package “glmnet” was used to perform the LASSO regression analysis (Friedman et al., 2010) on the training cohort, and overfitting was prevented by tenfold cross-validation. Lastly, according to the LASSO regression coefficients, the scoring system was constructed, which prognostic grouping was performed accordingly. With the help of the “survival” package in R, we compared the overall survival of both groups. To evaluate the stability of the model, we performed ROC curves and calculated AUC for different survival times and different clinical traits using the “survival” package. Key genes were obtained by intersecting differentially expressed m5C-related regulators from the TCGA and GEO data set, and prognosis-related genes from our prognostic model. Afterwards, we validated the expression of key genes in different subgroups. Supplementary Figure S1 shows the technology roadmap of the study.

On the basis of risk scores and clinical characteristics, we constructed a nomogram for predicting HCC patients’ survival probabilities. Afterwards, the discriminative power of the nomogram was measured by calibration curve and C-index value obtained from bootstrap analysis (1,000 replicates). The interactive nomogram was drawn using the R package “regplot”.

### GenSet enrichment analysis and gene set variation analysis enrichment analysis

Gene Set Enrichment Analysis (GSEA) allows us to examine the distribution of genes within predefined gene sets in a gene list which arranged according to their phenotype correlation, and thus determine how they contribute to the phenotype (Subramanian et al., 2005). The MSigDB database (http://www.gsea-msigdb.org/gsea/index.jsp) provided “c2.kegg.v7.4.symbols” and “c5.go.v7.4.symbols” gene sets (Liberzon et al., 2015). The R package “clusterprofiler” (Yu et al., 2012) can be used to perform GSEA analysis for those two gene sets in high and low-risk groups, where a *p* value less than 0.05 qualifies as statistically significant.

Gene Set Variation Analysis (GSVA) is a non-parametric, unsupervised method for evaluating gene set enrichment in transcriptomes. Through the conversion expression matrices of genes into expression matrices of gene sets, it is possible to assess the enriched metabolic pathways in different samples. GSVA analyses on the two gene sets mentioned above in different groups was conduct with “GSVA” package (Hänzelmann et al., 2013) and visualized using the “pheatmap” package.

### Immune infiltration in hepatocellular carcinoma

By using gene expression profiles, ESTIMATE R package predicted stromal and immune cell scores, and calculated their numbers for the analysis of HCC tumor purity in this study. We further compared the ESTIMATE scores among cancer and para-cancer groups, and among high and low-risk groups.

### Molecular isoform construction

Based on “ConsensusClusterPlus” package (Wilkerson and Hayes, 2010) we clustered cancer and para-cancer samples from TCGA and GEO databases into different groups by m5C regulator genes expression in each sample. The parameters were set to 50 replicates and a resampling rate of 80% (pItem = 0.8). To determine the validity of the groupings, a PCA was carried out, and the results were plotted using the “ggplot2” package. We also analyzed the correlation between prognostic models, molecular subtypes, and clinicopathological features based on TCGA data. Additionally, we examined the correlation between different subgroups and risk scores, and the expression of key genes in different subgroups.

### Immune infiltration analysis

CIBERSORT is a deconvolution algorithm that utilizes linear support vector regression to evaluate the expression matrices of immunocellular subtypes, and now is increasingly being used for immune infiltration characterization analysis in non-tumor tissues (Ge et al., 2021). Infiltration analysis of immune cells in HCC patients using RNA-Seq data can be an important guide in disease research and treatment prognosis prediction, etc. (Newman et al., 2019). With the CIBERSORT algorithm, this study compared immune cell infiltration levels between different prognostic model subgroups and different molecular subtype groupings, to examine how immune cells infiltration relates to different models.

### Molecular isotype construction of immune checkpoint genes

Immune checkpoint genes were obtained from a review [34]. We clustered cancer and para-cancer samples of the TCGA by the expression level of immune checkpoint genes using the R package “ConsensusClusterPlus” with 50 repetitions and a pItem = 0.8. To determine the validity of the grouping. PCA was used to analyze the genes expression levels, and “ggplot2” package visualize the results. The expression of key m5C regulator genes was also assessed in different subgroups. Using correlation analysis, we examined whether key m5C regulator genes play a role in HCC through immune cell infiltration.

### Statistical analysis

R version 4.0.2 was used for calculations and statistical analysis (https://www.r-project.org). Student’s t-tests (normally distributed variables) and Mann-Whitney U-tests (nonnormally distributed variables) were used for the comparison of continuous variables between two groups. All statistical *p* values all had a two-sided significance with *p* < 0.05.

## Results

### Expression characteristics of m5C regulator genes in hepatocellular carcinoma

**FIGURE 1:**
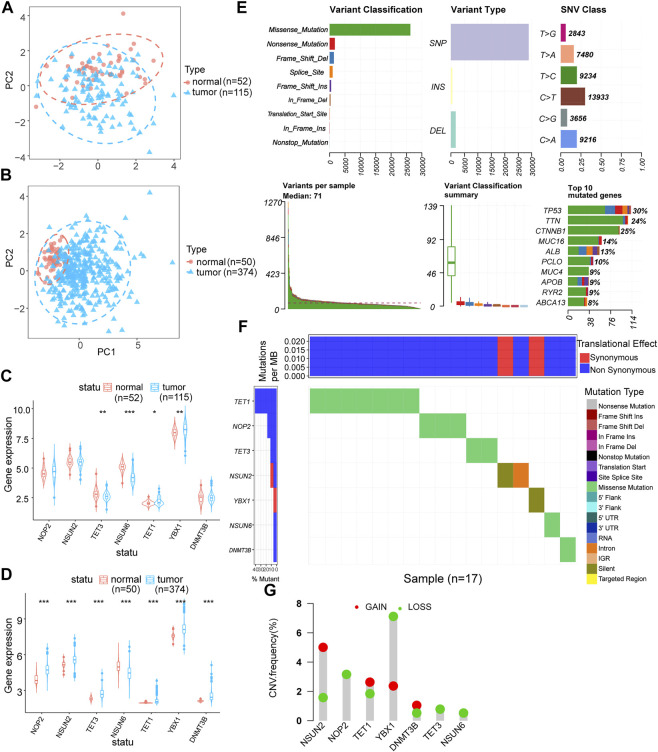
m5C regulator genes analysis. **(A,B)**: PCA analysis of GEO and TCGA expression matrices after data correction, blue represents tumor samples (GEO: *n* = 115, TCGA: *n* = 374) and red represents control samples (GEO: *n* = 52, TCGA: *n* = 50); **(C,D)**: differential expression analysis of m5C regulator genes in GEO and TCGA expression matrices after data correction, blue represents tumor samples (GEO: *n* = 115, TCGA: *n* = 374) and red represents control samples (GEO: *n* = 52, TCGA: *n* = 50); **(E)**: mutation profile of m5C regulator genes in hepatocellular carcinoma; **(F)**: m5C-related regulators SNV mutation category and frequency; **(G)**: m5C regulator genes CNV amplification and deletion.

We performed PCA analysis on the corrected datasets from GEO and TCGA, the results suggested a good correction effect (Figures 1A,B). Referring to Cui et al.'s study (Cui et al., 2021; Wang et al., 2021; Liu et al., 2022a), we selected the seven most common m5C regulator genes (NOP2, NSUN2, TET3, NSUN6, TET1, YBX1, and DNMT3B) as the subjects. In the GEO dataset, four of the seven m5C regulator genes (TET3, NSUN6, TET1, and YBX1) were differentially expressed (Figure 1C), while all seven m5C regulator genes had significant differential expression in the TCGA dataset (Figure 1D). Figure 1E lists the overall m5C regulator genes SNP

mutations in HCC samples situation, and Figure 1F shows the mutation types of different m5C regulator genes most closely associated with the development of HCC. We used CNV data from TCGA to identify significantly missing or amplified m5C regulator genes. Among the m5C regulator genes, YBX1 had the highest deletion frequency and the lowest amplification frequency (Figure 1G).

### Construction of prognostic model of m5C regulator genes and screening of key m5C regulator genes

**FIGURE 2:**
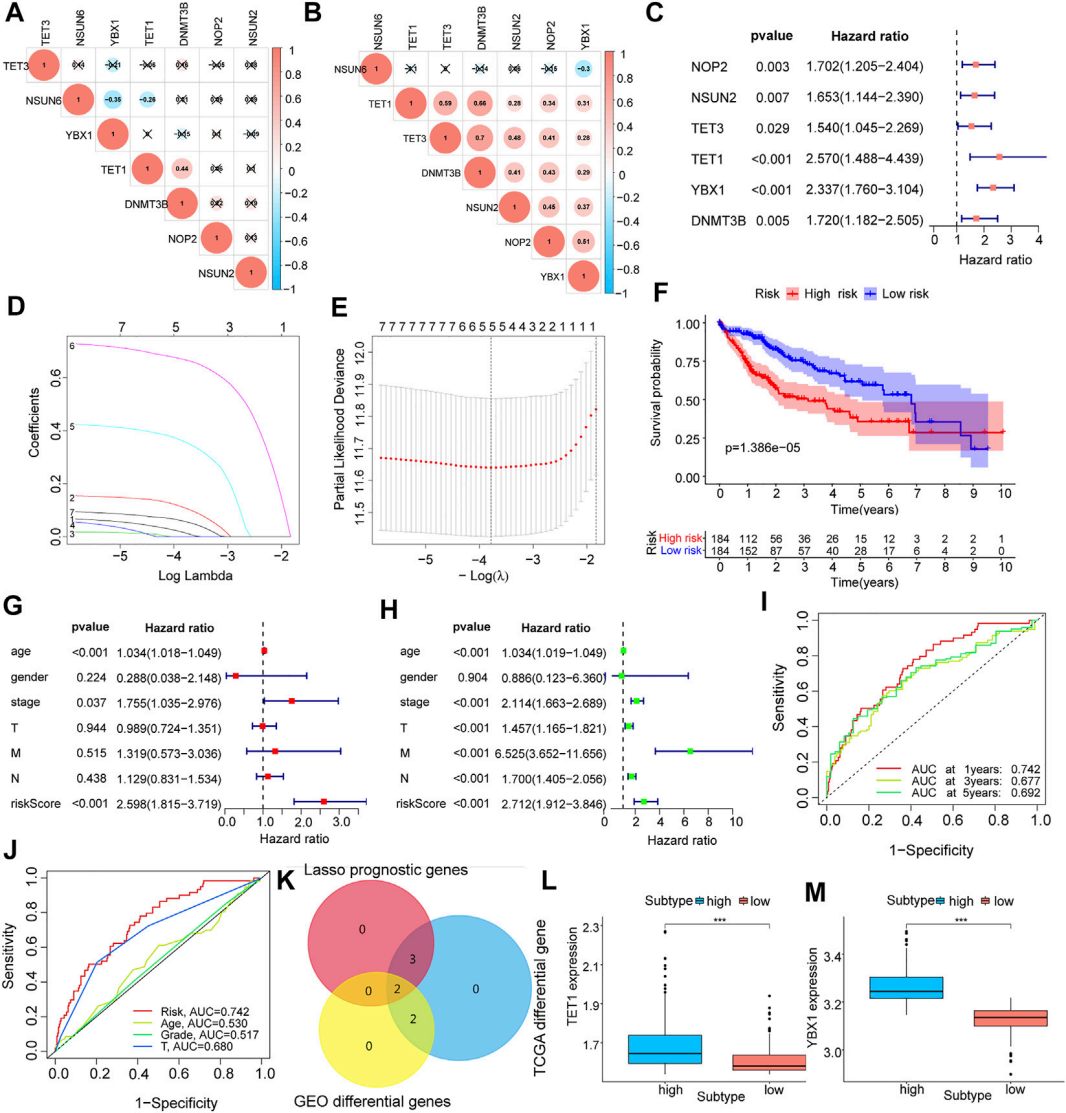
Expression characteristics and prognostic model construction of m5C regulator genes in hepatocellular carcinoma. **(A,B)**: m5C regulator genes co-expression analysis in the corrected GEO **(A)** and TCGA **(B)** expression matrices; **(C)**: identify m5C regulator genes associated with prognosis by univariate COX regression analysis, forest plots show the screened genes; **(D,E)**: show the regression coefficients in the LASSO regression algorithm and the cross-validation in the proportional risk model to adjust the parameter, finalize the best parameter(λ) to screen the most relevant genes for hepatocellular carcinoma; **(F)**: survival analysis of different LASSO subgroups; **(G,H)**: multivariate and univariate analysis of risk scores combined with clinical factors such as patient age, gender, and TNM stage; **(I,J)**: AUC analysis of prognostic model and clinical characteristics; **(K)**: Venn diagram mapping of differential genes in GEO and TCGA liver cancer samples and the intersection of genes screened out by LASSO; **(L,M)**: expression of key genes TET1 and YBX1 in each LASSO subgroups.

**FIGURE 3:**
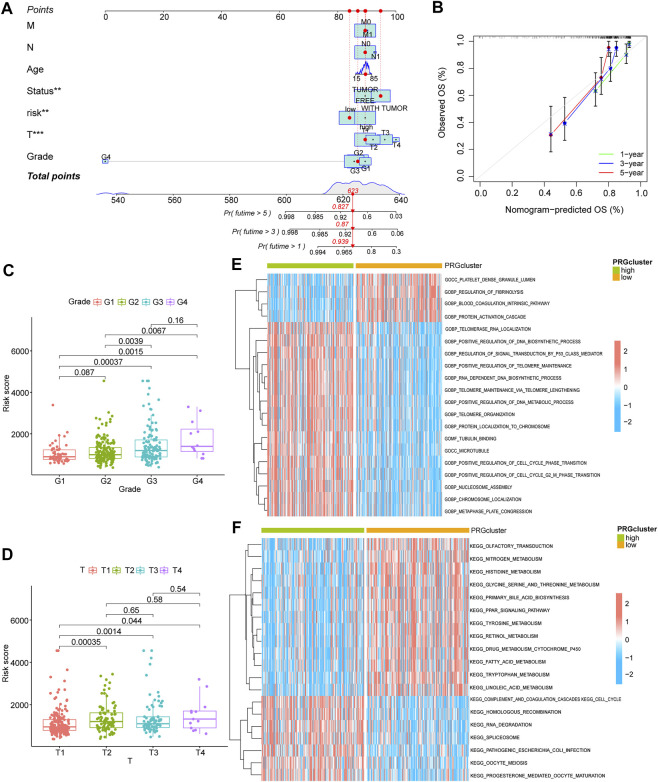
Clinical analysis and GSVA analysis of the prognostic model. **(A)** risk score combined with clinical indicators to draw nomogram; **(B)** comparison of predicted survival time and actual survival time using nomogram; **(C,D)**: correlation analysis for G-stage and T-stage, respectively; **(E,F)**: GSVA-GO analysis **(E)** and GSVA-KEGG analysis **(F)** for high and low-risk group.

Using co-expression analysis (Figures 2A,B) and univariate COX regression analysis (Figure 2C; Table 2), we assessed the effects of m5C regulator genes on HCC tissues. In co-expression analysis, TET1 and DNMT3B showed a significant positive correlation, and regression analysis screened six genes, including NOP2, NSUN2, TET3, TET1, YBX1, and DNMT3B, were associated with HCC. We constructed a LASSO prognostic model containing five genes, including NOP2, NSUN2, TET1, YBX1, and DNMT3B (Figures 2D,E), and a median risk score was used to separate HCC patients into two groups. It was demonstrated that low-risk patients lived significantly longer (Figure 2F). We evaluated COX regressions based on risk scores and clinical traits (age, gender, and TNM stage) using univariate and multivariate models (Figures 2G,H). Using AUC, we validated the LASSO prognostic model, and demonstrated that risk scores were highly predictive for 1-year, 3-years, and 5-years survival (Figures 2I,J). To further screen the key m5C regulator genes, we performed an intersection between DEGs from GEO and TCGA dataset and the key genes identified by LASSO modal, and finally obtained two of them, TET1, and YBX1 (Figure 2K), and it suggested that both two genes were higher expressed in high-risk group (Figures 2L–M). In combination with risk scores and clinical information, a nomogram (Figure 3A) and its calibration curve were constructed (Figure 3B), and we observed that sample’s risk scores tended to increase with the progression of T-stage and grade (Figures 3C,D), which is consistent with our previous predictions.

**TABLE 2:** Univariate Cox regression analysis.

Id	HR	HR.95L	HR.95H	*p*-value
NOP2	1.70	1.21	2.40	2.54E-03
NSUN2	1.65	1.14	2.39	7.47E-03
TET3	1.54	1.05	2.27	2.90E-02
NSUN6	0.92	0.71	1.20	5.60E-01
TET1	2.57	1.49	4.44	7.12E-04
YBX1	2.34	1.76	3.10	4.52E-09
DNMT3B	1.72	1.18	2.50	4.63E-03

### Evaluation of prognostic model for m5C regulator genes

We performed a GSVA analysis of the molecular functions for the different groups classified by the LASSO model. Low-risk group focused on functions relating to platelet dense granule lumen, regulation of fibrinolysis, blood coagulation intrinsic pathway, and protein activation cascade according to GO analysis (Figure 3E; Supplementary Table S1). KEGG analysis revealed it focused on olfactory transduction, nitrogen metabolism, histidine metabolism, serine and threonine metabolism (Figure 3E; Supplementary Table S1). We also performed GSEA analysis (Supplementary Figures S1A–D, Supplementary Table S2). As shown by GO analysis, the high-risk group was related to functions such as actin filament organization, actin polymerization or depolymerization, adaptive immune response, αβT cell activation, and anatomical structure homeostasis (Supplementary Figure S1A), while the low-risk group was linked to functions such as bile acid secretion, drug transmembrane transport, fatty acid *β* oxidation using acyl-CoA dehydrogenase, negative regulation of triglyceride metabolic process, and neurotransmitter catabolic process (Supplementary Figure S1B). According to KEGG analysis, pathways of high-risk group appeared to be enriched in Chemokine signaling pathway, cell adhesion molecules cams, Cell cycle, spliceosome, and Fc gamma r mediated phagocytosis (Supplementary Figure S1C). For low-risk group, pathways were enriched in beta alanine metabolism, histidine metabolism, linoleic acid metabolism, primary bile acid biosynthesis, and renin angiotensin system (Supplementary Figure S1D). We scored each subgroup using the ESTIMATE algorithm, and found a higher immune score in the high-risk group (Supplementary Figure S1E), but a lower stromal score, immune score, and ESTIMATE total score in the tumor group (Supplementary Figure S1F).

### Molecular typing of m5C regulator genes and correlation analysis

**FIGURE 4:**
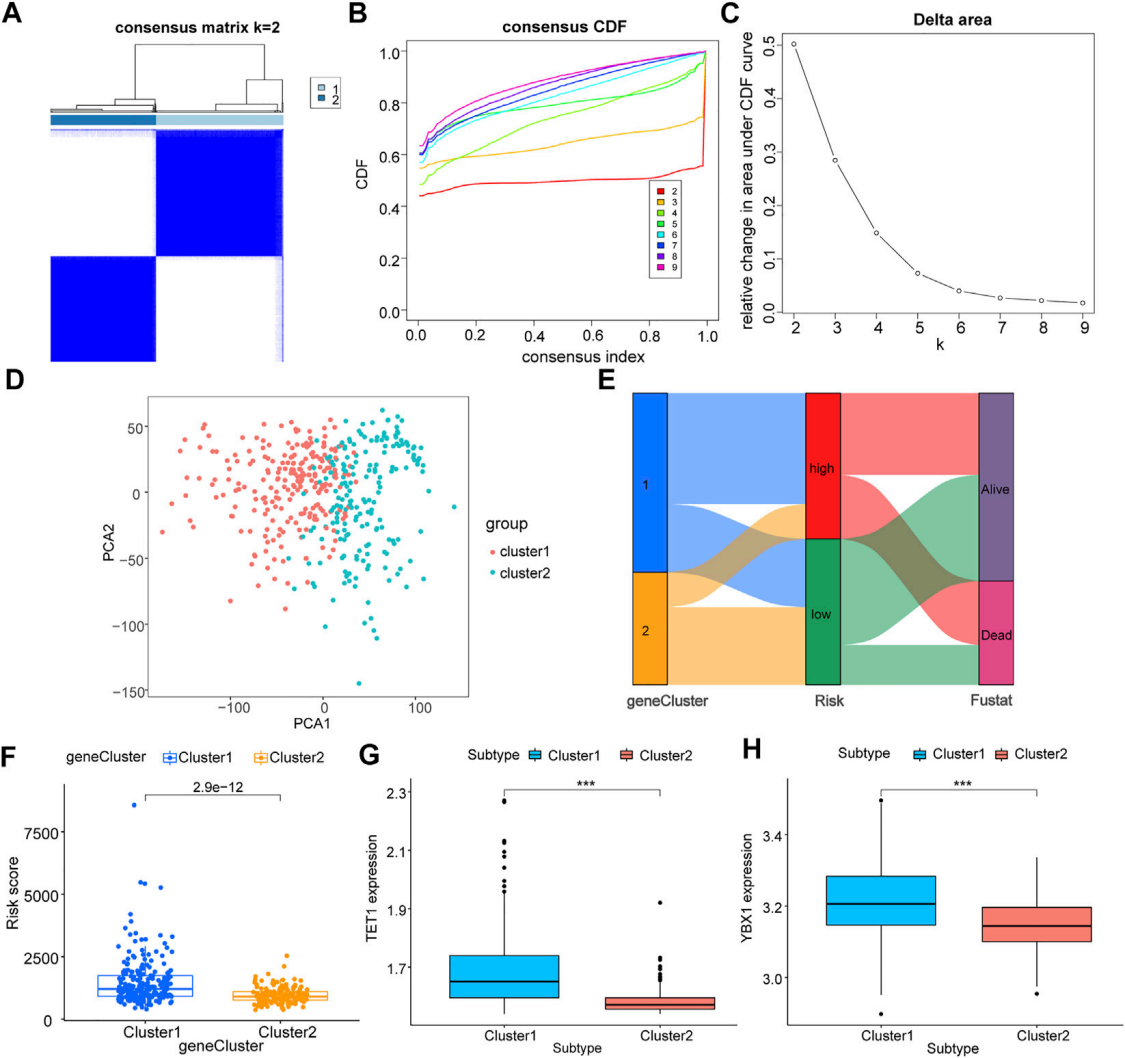
Correlation analysis of m5C regulator genes with molecular subtypes of TCGA liver cancer. All samples of TCGA were clustered according to their expression level of m5C regulator genes; **(A)**: sample size after grouping; **(B)**: change in area under the CDF curve (*k* = 2–9); **(C)**: change of delta area plot when *k* = 2 to *k* = 9; D: PCA analysis of cluster1 and cluster2, where cluster 1 is in red and cluster 2 is in blue; **(E)**: Sankey diagram combining survival status and LASSO model grouping; **(F)**: difference in risk scores of different groupings, cluster 1 in blue and cluster 2 in orange; **(G,H)**: differential expression of key m5C regulator genes TET1 **(G)** and YBX1 **(H)** in different groupings, cluster 1 in blue and cluster 2 in red.

In an effort to a better understood for the biological characteristics of m5C regulator genes in HCC patients, TCGA samples were clustered according to their expression level. Two subtypes of samples were identified (1: *n* = 232; 2: *n* = 192, Figures 4A–C), which PCA result showed high separation quality (Figure 4D), and in combination with the survival information of HCC patients and the grouping information of LASSO model, we constructed a Sankey diagram (Figure 4E). Cluster1 shows a significantly higher risk score compared to cluster2 (Figure 4F), confirming again the previous results. The differential analysis indicated the two key genes, TET1 and YBX1 were significantly higher expressed in cluster1 (*p* < 0.05, Figures 4G,H).

**FIGURE 5:**
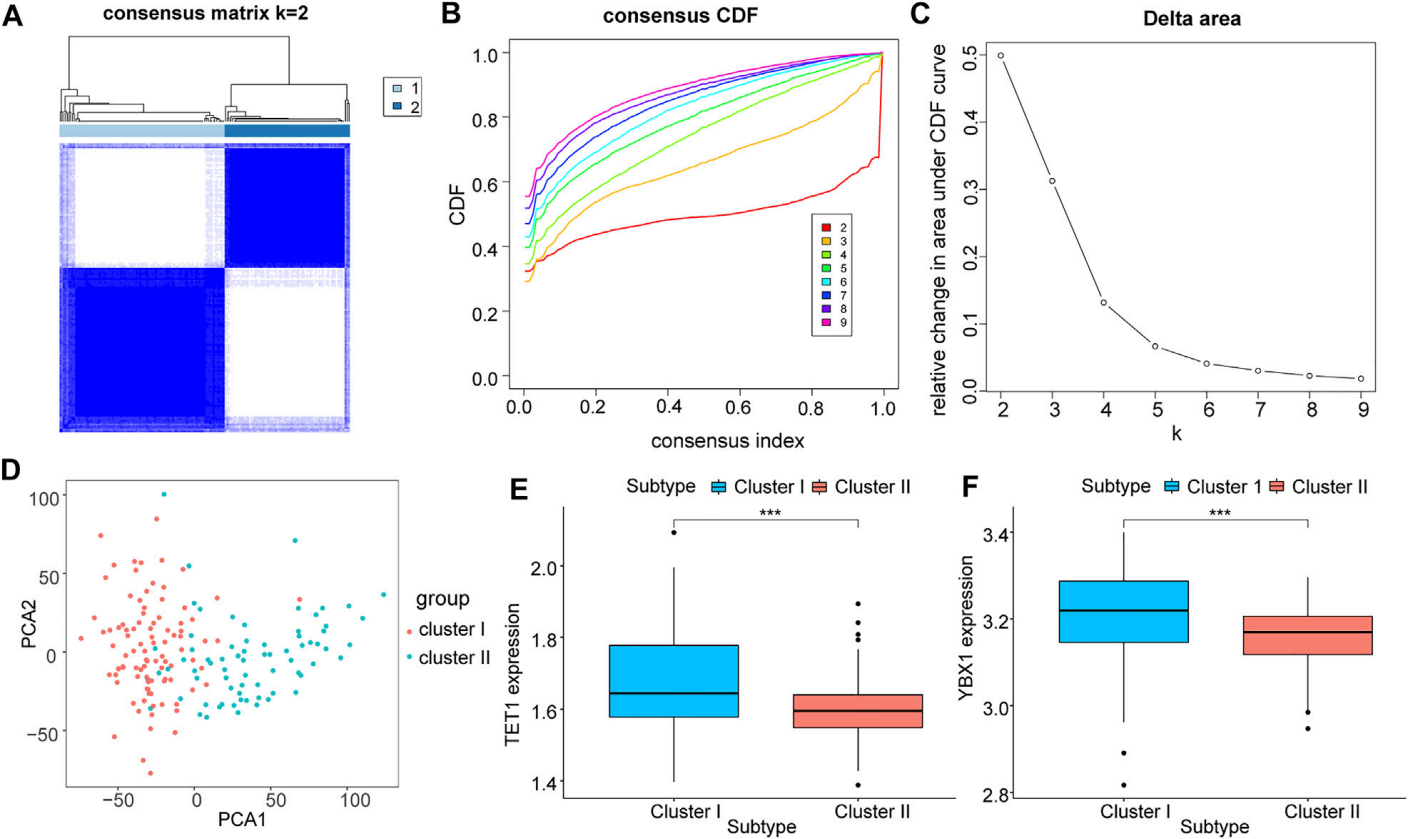
Correlation analysis of m5C regulatory genes with molecular subtypes of GEO liver cancer. **(A-C)**: All samples of GEO were classified by the expression level of m5C regulator genes; **(D)**: PCA analysis under different groupings; **(E,F)**: differential expression of key m5C regulator genes TET1 **(E)** and YBX1 **(F)** in different groupings, where cluster I is in blue and cluster II is in red.

We validated the previous results using the GEO expression matrix and samples were also classified into two isoforms (I: *n* = 95; 2: II = 72, Figures 5A–C). PCA result showed a higher quality of isolation (Figure 5D), and above two key genes were also present in cluster I with significantly higher expression (*p* < 0.05, Figures 5E,F).

### Correlation analysis between m5C regulator genes and immune infiltration

**FIGURE 6:**
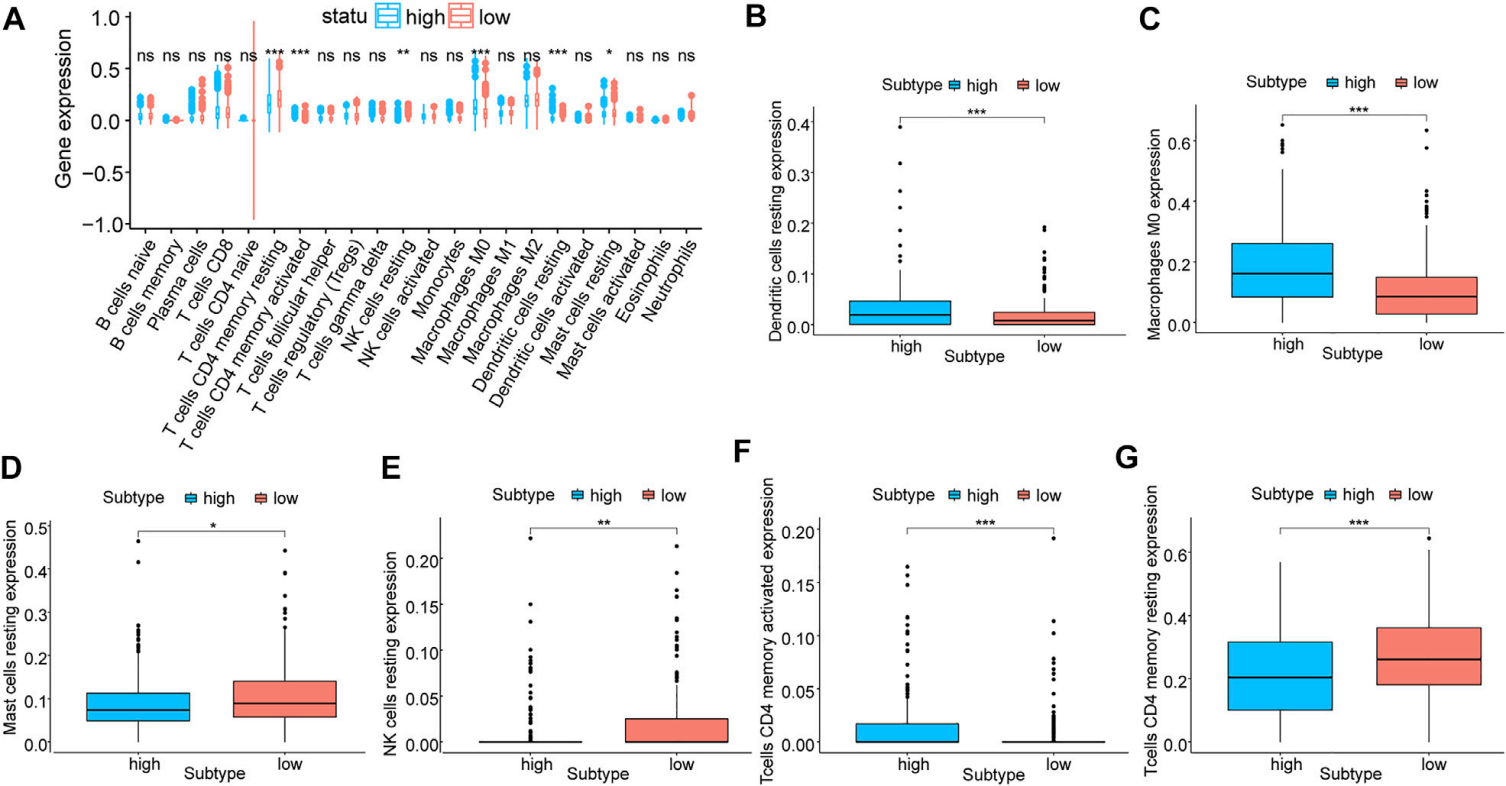
Correlation analysis of prognostic models and immune cells. **(A)** Differential analysis of the degree of immune cell infiltration in prognostic model subgroups, blue for high-risk and red for low-risk; **(B–G)**: Differential analysis of the degree of infiltration of six types of immune cells, including resting dendritic cells, M0 Macrophages, resting mast cells, resting NK cells, activated CD4 T cells, and resting CD4 T cells.

Through CIBERSORT, we calculated the infiltration degree of 22 immune cell types in two groups classified by the LASSO model to compare their variability of immune infiltration. A significant difference was observed in the infiltration degree of six kinds of immune cells when using the wilcox.test algorithm (Figure 6), namely activated CD4 T cells、resting CD4 T cells, resting NK cells, M0 Macrophages, resting dendritic cells, and resting mast cells. Among them, four immune cells types were *p* < 0.001, one kind was *p* < 0.01, and another kind was *p* < 0.05. Additionally, nine kinds of immune cells showed a difference in their infiltration degree between two subtypes of molecular typing (Supplementary Figure S2), namely activated CD4 T cells, T gamma delta cells, naive B cells, M0 Macrophages, resting CD4 T cells, Monocytes, M2 Macrophages, T follicular helper cells, and Tregs cells, and six of them were *p* < 0.001, one was *p* < 0.01 and two were *p* < 0.05.

### Molecular isotype construction of immune checkpoint genes

**FIGURE 7:**
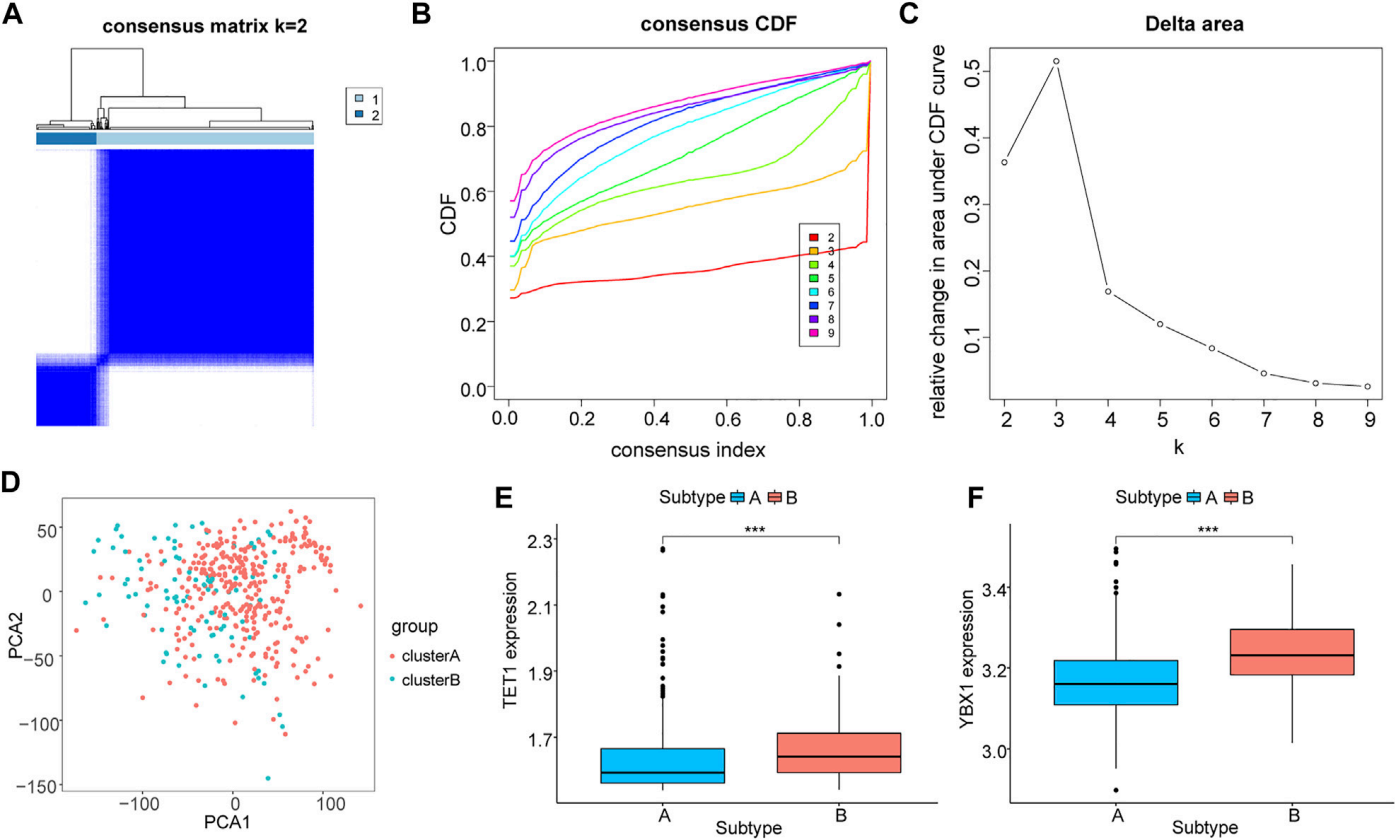
Molecular typing based on immune checkpoint genes. **(A–C)**: Cluster grouping based on immune checkpoint genes, **(A)**: sample size after grouping; **(B)**: change in area under the CDF curve (*k* = 2–9); **(C)**: change of delta area plot when *k* = 2 to *k* = 9; **(D)**: PCA analysis of cluster A and B; E-F: differential expression level of TET1 **(E)** and YBX1 **(F)** in different groupings, cluster **(A)** in blue and cluster **(B)** in red.

We used significantly differentially express immune checkpoint genes to conduct hierarchical clustering of all HCC samples again to find out the correlation between these genes and m5C. Among all samples, two subtypes were identified (A: *n* = 332; B: *n* = 92, Figures 7A–C). The PCA result showed a high quality of separation (Figure 7D), and differential analysis showed that TET1 and YBX1 were significantly differentially expressed in different subgroups (*p* < 0.01, Figures 7E,F.

### Correlation analysis between key m5C regulator genes and immune cells

Using correlation analysis between key m5C regulator genes and the immune microenvironment, we examined the potential correlation between m5C regulators and immunotherapy efficacy. Combining CIBERSORT results with key m5C regulator genes, we found a positive correlation between TET1 and the infiltration level of various kinds of innate or acquired immune cells, such as M0 macrophages, resting dendritic cells, and T follicular helper cells, while a significant negative correlation with M1 macrophages, M2 macrophages, and resting mast. There was a positive correlation between YBX1 and resting dendritic cells and M0 macrophages, but a negative correlation with Tregs and CD4 T cells (Supplementary Figure S3).

## Discussion

Ongoing studies have showed that RNA modification contributes to tumorigenesis and tumor progression, and there is growing evidence that m5C regulator genes may serve as potential biomarkers for cancer prediction (Huang et al., 2021a; Huang et al., 2021b; Cui et al., 2021; Xue et al., 2021). It has been suggested that 5 mC methylation influences the development of HCC including clinical stage, progression, and prognosis (Villanueva et al., 2015; Hlady et al., 2019), but the relationship between m5C-related RNA modification and HCC is still poorly understood. In order to test whether these genes can provide prognostic clues for HCC and assist in its initiation and progression, we need to focus on their aberrant expression in HCC. This study confirmed that m5C regulator genes was differentially expressed between HCC and normal samples.

The difference of m5C regulator genes expression levels between tumor and paraneoplastic tissues suggested that these genes may be associated with the carcinogenesis and progression of HCC. MeRIP-seq was used in one study to analyze the m5C modification in tumor and paraneoplastic tissues, and it was found that m5C modification peaks were more abundant and higher in mRNA of HCC tissues, which reconfirmed the relevance of m5C in this disease (Zhang et al., 2020). Aberrant gene methylation is strongly associated with HCC, both in frequency and amount (Nishida et al., 2008).

We constructed a LASSO regression model, which showed satisfactory predictive performance. Similarly, He et al. (2020b) utilized TCGA data developed a two-gene signature of m5C regulators (NSUN4 and ALYREF) with HCC prognostic value based on the LASSO and multivariate Cox regression models. Also demonstrate that the role of m5C related regulators in HCC are dysregulated and associated with patient survival. The methodology we used is largely similar, the major difference being is that we analyzed GEO data combined with the TCGA analysis. In fact, our study proves that utilizing multiple datasets and analytic approaches may identify important gene signatures that would otherwise not be identified using a single dataset/approach. Ultimately this may improve the validity of the findings and be a stronger indication to evaluate these genes in experimental and clinical settings.

For a comprehensive analysis, we performed GSVA and GSEA analyses. “Adaptive immune response” and “cell cycle” et al. are found related to hepatocarcinogenesis and progression. M5C-related RNA modifications impact mRNA translation, transport, and stability, and m5C regulator genes appeared to be associated with “spliceosomes” in this study, suggesting their importance in RNA processing.

Tumor cells are the drivers of tumor development, but they can’t function alone during tumor progression without the tumor microenvironment (TME). Blood vessels, fibroblasts, immune cells, extracellular matrix, and signaling molecules are all components of the TME which contribute to tumorigenesis and tumor progression. Evidence suggests m5C-related regulators are associated with the tumor immune microenvironment (Geng et al., 2021). Numerous tumors have been studied to correlate tumor immune cell infiltration with clinical outcome (Ishigami et al., 2000; Villegas et al., 2002; Hamanishi et al., 2007; Sharma et al., 2007; Zhu et al., 2009; Mahmoud et al., 2011), however, we do not yet know how m5C modification affects the immune system in HCC. Here, we describe the infiltration characteristics of TME cells in different model groupings and perform immune scoring, which shed light on the molecular mechanism of HCC and new clues for prognosis prediction.

As a result of its aggressiveness, metastasis, and refractoriness, HCC has a high mortality rate and poor prognosis (Ioannou, 2021). While medical technology continues to advance and therapeutic approaches vary, there are still no ideal therapeutic targets or targeted interventions for HCC because its molecular mechanisms of carcinogenesis and development are still unclear (Jiří et al., 2020). It has been shown that azacytidine can reduce cancer cells proliferation by inhibiting m5C modification (Esteller and Pandolfi, 2017), suggesting that reducing m5C modification may contribute to cancer treatment. Ultimately, different RNA epigenetic modifications mediated by regulatory factors provide new idea for finding potential therapeutic targets.

From the perspective of combined multi-omics analysis, we explored the expression profiling of m5C-related genes in HCC, correlation prognostic model construction and evaluation, molecular typing and correlation analysis, immune cell infiltration correlation analysis, immune checkpoint gene molecular subtype construction, and immune cell correlation analysis. Other functions, limited by the length of this study, we really did not study, but we intend to verify other biological functions of m5C through the experimental perspective by doing experiments such as WB, PCR and IHC.

## Conclusion

The study combined data from TCGA and GEO for the first time to reveal the genetic and prognostic significance of m5C-related regulators in HCC, which provides new directions for identifying predictive biomarkers and developing molecularly targeted therapies for HCC.

## Data Availability

The original contributions presented in the study are included in the article/Supplementary Material, further inquiries can be directed to the corresponding author.
