# Supplementary material for: Diagnostic and prognostic value of m5C regulatory genes in hepatocellular carcinoma
Source: Front Genet. 2022 Aug 29;13:972043. doi: 10.3389/fgene.2022.972043 (PMC9465290; doi:10.3389/fgene.2022.972043)
Supplement: Supplementary file 1 [file DataSheet1.docx]

Supplementary Material

**Figure S1: The technology roadmap of the study**

**Figure S2: GSEA analysis and immune infiltration analysis of the prognostic model**

A: GSEA-GO analysis results of high-risk group, B: GSEA-GO analysis results of low-risk group, C: GSEA-KEGG analysis results of high-risk group, D: GSEA-KEGG analysis results of low-risk group; E: Stromal score, immune score and total score of different risk groups, F: Stromal score, immune score and total score of tumor and control group.

**Figure S3: Correlation analysis of molecular typing and immune cells**

A: Differential analysis of the degree of immune cell infiltration in different molecular subtype groupings, cluster1 in blue and cluster2 in red; B-J: Differential analysis of the degree of infiltration of 9 types of immune cells, including naive B cells, M0 Macrophages, M2 Macrophages, Monocytes, activated CD4 T cells, resting CD4 T cells, T follicular helper cells, T gamma delta cells, and Tregs cells.

**Figure S4: Correlation analysis of key m5C regulator genes and immune cells**

Correlation analysis of key m5C regulator genes with immune cells, including M0 macrophages, resting dendritic cells, M1 macrophages, M1 macrophages, and resting mast cells et al, the slope is the size of correlation and P-value indicates the level of significance.

**Table S**1 GSVA analysis

| **id** | **logFC** | **adj.P.Val** |
| --- | --- | --- |
| GOBP_BLOOD_COAGULATION_INTRINSIC_PATHWAY | 0.46 | 4.59E-24 |
| GOBP_DOUBLE_STRAND_BREAK_REPAIR_VIA_BREAK_INDUCED_REPLICATION | -0.38 | 3.67E-14 |
| GOBP_ESTABLISHMENT_OF_PROTEIN_LOCALIZATION_TO_TELOMERE | -0.38 | 4.81E-22 |
| GOBP_FATTY_ACID_BETA_OXIDATION_USING_ACYL_COA_DEHYDROGENASE | 0.42 | 8.32E-16 |
| GOBP_NEGATIVE_REGULATION_OF_FIBRINOLYSIS | 0.40 | 1.49E-19 |
| GOBP_POSITIVE_REGULATION_OF_TELOMERASE_RNA_LOCALIZATION_TO_CAJAL_BODY | -0.39 | 4.95E-19 |
| GOBP_PROTEIN_ACTIVATION_CASCADE | 0.40 | 1.48E-22 |
| GOBP_REGULATION_OF_ATTACHMENT_OF_SPINDLE_MICROTUBULES_TO_KINETOCHORE | -0.39 | 5.69E-22 |
| GOBP_REGULATION_OF_ESTABLISHMENT_OF_PROTEIN_LOCALIZATION_TO_CHROMOSOME | -0.41 | 7.11E-18 |
| GOBP_REGULATION_OF_FIBRINOLYSIS | 0.43 | 1.48E-22 |
| GOBP_REGULATION_OF_PROTEIN_LOCALIZATION_TO_CHROMOSOME_TELOMERIC_REGION | -0.42 | 2.68E-19 |
| GOBP_TELOMERASE_RNA_LOCALIZATION | -0.39 | 3.34E-22 |
| GOCC_CHYLOMICRON | 0.40 | 4.78E-16 |
| GOCC_CMG_COMPLEX | -0.40 | 3.56E-14 |
| GOCC_DNA_REPLICATION_PREINITIATION_COMPLEX | -0.40 | 3.56E-14 |
| GOMF_ALCOHOL_DEHYDROGENASE_NAD_P_PLUS_ACTIVITY | 0.42 | 1.68E-14 |
| GOMF_ARACHIDONIC_ACID_EPOXYGENASE_ACTIVITY | 0.41 | 9.93E-17 |
| GOMF_ARACHIDONIC_ACID_MONOOXYGENASE_ACTIVITY | 0.41 | 5.74E-17 |
| GOMF_AROMATASE_ACTIVITY | 0.41 | 2.22E-20 |
| GOMF_DNA_REPLICATION_ORIGIN_BINDING | -0.38 | 3.16E-17 |
| KEGG_BASE_EXCISION_REPAIR | -0.17 | 7.77E-08 |
| KEGG_CELL_CYCLE | -0.26 | 1.63E-20 |
| KEGG_COMPLEMENT_AND_COAGULATION_CASCADES | 0.36 | 7.14E-19 |
| KEGG_DNA_REPLICATION | -0.28 | 1.09E-11 |
| KEGG_DRUG_METABOLISM_CYTOCHROME_P450 | 0.33 | 3.90E-17 |
| KEGG_FATTY_ACID_METABOLISM | 0.36 | 1.11E-15 |
| KEGG_FC_GAMMA_R_MEDIATED_PHAGOCYTOSIS | -0.17 | 3.80E-11 |
| KEGG_GLYCINE_SERINE_AND_THREONINE_METABOLISM | 0.35 | 9.52E-16 |
| KEGG_HISTIDINE_METABOLISM | 0.30 | 1.48E-18 |
| KEGG_HOMOLOGOUS_RECOMBINATION | -0.28 | 2.70E-15 |
| KEGG_LINOLEIC_ACID_METABOLISM | 0.30 | 1.63E-20 |
| KEGG_MISMATCH_REPAIR | -0.25 | 2.65E-12 |
| KEGG_NON_HOMOLOGOUS_END_JOINING | -0.20 | 3.10E-09 |
| KEGG_PATHOGENIC_ESCHERICHIA_COLI_INFECTION | -0.20 | 2.68E-17 |
| KEGG_PRIMARY_BILE_ACID_BIOSYNTHESIS | 0.42 | 6.83E-17 |
| KEGG_RETINOL_METABOLISM | 0.34 | 3.32E-17 |
| KEGG_RNA_DEGRADATION | -0.23 | 7.59E-18 |
| KEGG_SPLICEOSOME | -0.27 | 5.73E-16 |
| KEGG_TRYPTOPHAN_METABOLISM | 0.30 | 5.02E-15 |
| KEGG_VALINE_LEUCINE_AND_ISOLEUCINE_DEGRADATION | 0.30 | 1.50E-11 |

**Table S2** GSEA analysis

| **Description** | **Enrichment Score** | **NES** | **p.adjust** |
| --- | --- | --- | --- |
| GOBP_ACTIN_FILAMENT_ORGANIZATION | 0.43 | 1.29 | 1.87E-02 |
| GOBP_ACTIN_POLYMERIZATION_OR_DEPOLYMERIZATION | 0.45 | 1.34 | 1.87E-02 |
| GOBP_ADAPTIVE_IMMUNE_RESPONSE | 0.45 | 1.36 | 1.87E-02 |
| GOBP_ALPHA_BETA_T_CELL_ACTIVATION | 0.50 | 1.47 | 1.87E-02 |
| GOBP_ANATOMICAL_STRUCTURE_HOMEOSTASIS | 0.45 | 1.36 | 1.87E-02 |
| GOBP_ANTIGEN_PROCESSING_AND_PRESENTATION | 0.47 | 1.40 | 1.87E-02 |
| GOBP_ANTIGEN_PROCESSING_AND_PRESENTATION_OF_PEPTIDE_ANTIGEN | 0.46 | 1.36 | 1.87E-02 |
| GOBP_ANTIGEN_PROCESSING_AND_PRESENTATION_OF_PEPTIDE_OR_POLYSACCHARIDE_ANTIGEN_VIA_MHC_CLASS_II | 0.55 | 1.60 | 1.87E-02 |
| GOBP_ANTIGEN_RECEPTOR_MEDIATED_SIGNALING_PATHWAY | 0.47 | 1.42 | 1.87E-02 |
| GOBP_ATP_DEPENDENT_CHROMATIN_REMODELING | 0.61 | 1.74 | 1.87E-02 |
| GOBP_ATTACHMENT_OF_MITOTIC_SPINDLE_MICROTUBULES_TO_KINETOCHORE | 0.81 | 1.76 | 1.88E-02 |
| GOBP_ATTACHMENT_OF_SPINDLE_MICROTUBULES_TO_KINETOCHORE | 0.74 | 1.87 | 1.87E-02 |
| GOBP_B_CELL_ACTIVATION | 0.51 | 1.54 | 1.87E-02 |
| GOBP_B_CELL_ACTIVATION_INVOLVED_IN_IMMUNE_RESPONSE | 0.55 | 1.55 | 1.87E-02 |
| GOBP_B_CELL_DIFFERENTIATION | 0.49 | 1.45 | 1.87E-02 |
| GOBP_B_CELL_PROLIFERATION | 0.52 | 1.51 | 1.87E-02 |
| GOBP_B_CELL_RECEPTOR_SIGNALING_PATHWAY | 0.60 | 1.67 | 1.87E-02 |
| GOBP_CELL_ADHESION_MEDIATED_BY_INTEGRIN | 0.58 | 1.63 | 1.87E-02 |
| GOBP_CELL_CHEMOTAXIS | 0.48 | 1.46 | 1.87E-02 |
| GOBP_CELL_CYCLE_ARREST | 0.48 | 1.44 | 1.87E-02 |
| GOBP_CELL_CYCLE_CHECKPOINT | 0.55 | 1.64 | 1.87E-02 |
| GOBP_CELL_CYCLE_DNA_REPLICATION | 0.67 | 1.88 | 1.87E-02 |
| GOBP_CELL_CYCLE_G1_S_PHASE_TRANSITION | 0.53 | 1.61 | 1.87E-02 |
| GOBP_CELL_CYCLE_G2_M_PHASE_TRANSITION | 0.50 | 1.51 | 1.87E-02 |
| GOBP_CELL_MATURATION | 0.48 | 1.42 | 1.87E-02 |
| GOBP_CELL_PROJECTION_ASSEMBLY | 0.44 | 1.33 | 1.87E-02 |
| GOBP_CELL_SUBSTRATE_ADHESION | 0.44 | 1.33 | 1.87E-02 |
| GOBP_CELLULAR_RESPONSE_TO_HEAT | 0.49 | 1.44 | 1.87E-02 |
| GOBP_CENTROMERE_COMPLEX_ASSEMBLY | 0.74 | 1.93 | 1.87E-02 |
| GOBP_CHEMOKINE_PRODUCTION | 0.50 | 1.45 | 1.87E-02 |
| GOBP_CHROMATIN_ASSEMBLY_OR_DISASSEMBLY | 0.56 | 1.64 | 1.87E-02 |
| GOBP_CHROMATIN_ORGANIZATION_INVOLVED_IN_REGULATION_OF_TRANSCRIPTION | 0.52 | 1.48 | 1.87E-02 |
| GOBP_CHROMATIN_REMODELING | 0.55 | 1.62 | 1.87E-02 |
| GOBP_CHROMATIN_REMODELING_AT_CENTROMERE | 0.78 | 1.96 | 1.87E-02 |
| GOBP_CHROMOSOME_CONDENSATION | 0.71 | 1.85 | 1.87E-02 |
| GOBP_CHROMOSOME_LOCALIZATION | 0.59 | 1.68 | 1.87E-02 |
| GOBP_CHROMOSOME_ORGANIZATION_INVOLVED_IN_MEIOTIC_CELL_CYCLE | 0.62 | 1.70 | 1.87E-02 |
| GOBP_CHROMOSOME_SEGREGATION | 0.57 | 1.71 | 1.87E-02 |
| GOBP_CHROMOSOME_SEPARATION | 0.60 | 1.73 | 1.87E-02 |
| GOBP_CILIARY_BASAL_BODY_PLASMA_MEMBRANE_DOCKING | 0.53 | 1.51 | 1.87E-02 |
| GOBP_CORTICAL_ACTIN_CYTOSKELETON_ORGANIZATION | 0.64 | 1.69 | 1.87E-02 |
| GOBP_COVALENT_CHROMATIN_MODIFICATION | 0.45 | 1.38 | 1.87E-02 |
| GOBP_CYTOKINESIS | 0.47 | 1.37 | 1.87E-02 |
| GOBP_CYTOSKELETON_DEPENDENT_CYTOKINESIS | 0.51 | 1.47 | 1.87E-02 |
| GOBP_DNA_BIOSYNTHETIC_PROCESS | 0.48 | 1.43 | 1.87E-02 |
| GOBP_DNA_CONFORMATION_CHANGE | 0.54 | 1.61 | 1.87E-02 |
| GOBP_DNA_DAMAGE_RESPONSE_SIGNAL_TRANSDUCTION_BY_P53_CLASS_MEDIATOR | 0.54 | 1.57 | 1.87E-02 |
| GOBP_DNA_DEPENDENT_DNA_REPLICATION | 0.60 | 1.76 | 1.87E-02 |
| GOBP_DNA_GEOMETRIC_CHANGE | 0.51 | 1.50 | 1.87E-02 |
| GOBP_DNA_INTEGRITY_CHECKPOINT | 0.54 | 1.58 | 1.87E-02 |
| GOBP_DNA_PACKAGING | 0.59 | 1.73 | 1.87E-02 |
| GOBP_DNA_RECOMBINATION | 0.52 | 1.56 | 1.87E-02 |
| GOBP_DNA_REPAIR | 0.47 | 1.45 | 1.87E-02 |
| GOBP_DNA_REPLICATION | 0.55 | 1.64 | 1.87E-02 |
| GOBP_DNA_REPLICATION_INDEPENDENT_NUCLEOSOME_ORGANIZATION | 0.72 | 1.86 | 1.87E-02 |
| GOBP_DNA_REPLICATION_INITIATION | 0.78 | 2.01 | 1.87E-02 |
| GOBP_DNA_STRAND_ELONGATION | 0.76 | 1.91 | 1.87E-02 |
| GOBP_DNA_STRAND_ELONGATION_INVOLVED_IN_DNA_REPLICATION | 0.79 | 1.89 | 1.87E-02 |
| GOBP_DNA_UNWINDING_INVOLVED_IN_DNA_REPLICATION | 0.78 | 1.77 | 1.87E-02 |
| GOBP_DOUBLE_STRAND_BREAK_REPAIR | 0.51 | 1.52 | 1.87E-02 |
| GOBP_DOUBLE_STRAND_BREAK_REPAIR_VIA_BREAK_INDUCED_REPLICATION | 0.90 | 1.91 | 1.88E-02 |
| GOBP_EPIDERMAL_GROWTH_FACTOR_RECEPTOR_SIGNALING_PATHWAY | 0.50 | 1.45 | 1.87E-02 |
| GOBP_EPITHELIAL_TUBE_FORMATION | 0.47 | 1.39 | 1.87E-02 |
| GOBP_ESTABLISHMENT_OF_ORGANELLE_LOCALIZATION | 0.44 | 1.34 | 1.87E-02 |
| GOBP_ESTABLISHMENT_OF_RNA_LOCALIZATION | 0.51 | 1.52 | 1.87E-02 |
| GOBP_ESTABLISHMENT_OR_MAINTENANCE_OF_CELL_POLARITY | 0.45 | 1.33 | 1.87E-02 |
| GOBP_EXTERNAL_ENCAPSULATING_STRUCTURE_ORGANIZATION | 0.44 | 1.33 | 1.87E-02 |
| GOBP_FC_RECEPTOR_MEDIATED_STIMULATORY_SIGNALING_PATHWAY | 0.55 | 1.57 | 1.87E-02 |
| GOBP_FEMALE_MEIOTIC_NUCLEAR_DIVISION | 0.71 | 1.76 | 1.87E-02 |
| GOBP_G0_TO_G1_TRANSITION | 0.62 | 1.70 | 1.87E-02 |
| GOBP_GLIOGENESIS | 0.42 | 1.27 | 1.87E-02 |
| GOBP_GLYCINE_TRANSPORT | 0.81 | 1.80 | 1.87E-02 |
| GOBP_GRANULOCYTE_CHEMOTAXIS | 0.52 | 1.52 | 1.87E-02 |
| GOBP_GRANULOCYTE_MIGRATION | 0.52 | 1.51 | 1.87E-02 |
| GOBP_HISTONE_EXCHANGE | 0.69 | 1.83 | 1.87E-02 |
| GOBP_HOMEOSTASIS_OF_NUMBER_OF_CELLS | 0.45 | 1.36 | 1.87E-02 |
| GOBP_IMMUNE_RESPONSE_REGULATING_SIGNALING_PATHWAY | 0.48 | 1.45 | 1.87E-02 |
| GOBP_IMPORT_INTO_NUCLEUS | 0.47 | 1.39 | 1.87E-02 |
| GOBP_INTEGRIN_MEDIATED_SIGNALING_PATHWAY | 0.51 | 1.47 | 1.87E-02 |
| GOBP_INTERLEUKIN_6_PRODUCTION | 0.48 | 1.41 | 1.87E-02 |
| GOBP_INTERSTRAND_CROSS_LINK_REPAIR | 0.62 | 1.70 | 1.87E-02 |
| GOBP_INTRINSIC_APOPTOTIC_SIGNALING_PATHWAY | 0.43 | 1.31 | 1.87E-02 |
| GOBP_KINETOCHORE_ASSEMBLY | 0.77 | 1.68 | 1.88E-02 |
| GOBP_KINETOCHORE_ORGANIZATION | 0.84 | 1.97 | 1.87E-02 |
| GOBP_LEUKOCYTE_CELL_CELL_ADHESION | 0.50 | 1.50 | 1.87E-02 |
| GOBP_LEUKOCYTE_CHEMOTAXIS | 0.49 | 1.47 | 1.87E-02 |
| GOBP_LEUKOCYTE_DIFFERENTIATION | 0.47 | 1.42 | 1.87E-02 |
| GOBP_LEUKOCYTE_MIGRATION | 0.51 | 1.54 | 1.87E-02 |
| GOBP_LEUKOCYTE_PROLIFERATION | 0.48 | 1.45 | 1.87E-02 |
| GOBP_LYMPHOCYTE_ACTIVATION_INVOLVED_IN_IMMUNE_RESPONSE | 0.47 | 1.39 | 1.87E-02 |
| GOBP_MACROMOLECULE_METHYLATION | 0.43 | 1.29 | 1.87E-02 |
| GOBP_MACROPHAGE_ACTIVATION | 0.51 | 1.47 | 1.87E-02 |
| GOBP_MALE_GAMETE_GENERATION | 0.40 | 1.22 | 1.87E-02 |
| GOBP_MAST_CELL_ACTIVATION | 0.58 | 1.63 | 1.87E-02 |
| GOBP_MEIOSIS_I_CELL_CYCLE_PROCESS | 0.55 | 1.61 | 1.87E-02 |
| GOBP_MEIOTIC_CELL_CYCLE | 0.55 | 1.63 | 1.87E-02 |
| GOBP_MEIOTIC_CELL_CYCLE_PROCESS | 0.57 | 1.69 | 1.87E-02 |
| GOBP_MEIOTIC_CHROMOSOME_SEGREGATION | 0.58 | 1.65 | 1.87E-02 |
| GOBP_MEMBRANE_DOCKING | 0.49 | 1.44 | 1.87E-02 |
| GOBP_MEMBRANE_INVAGINATION | 0.57 | 1.60 | 1.87E-02 |
| GOBP_METAPHASE_ANAPHASE_TRANSITION_OF_CELL_CYCLE | 0.64 | 1.79 | 1.87E-02 |
| GOBP_METAPHASE_PLATE_CONGRESSION | 0.61 | 1.70 | 1.87E-02 |
| GOBP_MICROGLIAL_CELL_ACTIVATION | 0.62 | 1.69 | 1.87E-02 |
| GOBP_MICROTUBULE_CYTOSKELETON_ORGANIZATION | 0.48 | 1.48 | 1.87E-02 |
| GOBP_MICROTUBULE_CYTOSKELETON_ORGANIZATION_INVOLVED_IN_MITOSIS | 0.59 | 1.72 | 1.87E-02 |
| GOBP_MICROTUBULE_ORGANIZING_CENTER_ORGANIZATION | 0.49 | 1.44 | 1.87E-02 |
| GOBP_MITOTIC_CELL_CYCLE_CHECKPOINT | 0.59 | 1.72 | 1.87E-02 |
| GOBP_MITOTIC_CHROMOSOME_CONDENSATION | 0.76 | 1.72 | 1.87E-02 |
| GOBP_MITOTIC_DNA_INTEGRITY_CHECKPOINT | 0.56 | 1.63 | 1.87E-02 |
| GOBP_MITOTIC_DNA_REPLICATION | 0.79 | 1.76 | 1.87E-02 |
| GOBP_MITOTIC_G1_S_TRANSITION_CHECKPOINT | 0.59 | 1.64 | 1.87E-02 |
| GOBP_MITOTIC_METAPHASE_PLATE_CONGRESSION | 0.60 | 1.64 | 1.87E-02 |
| GOBP_MITOTIC_NUCLEAR_DIVISION | 0.56 | 1.69 | 1.87E-02 |
| GOBP_MITOTIC_SISTER_CHROMATID_SEGREGATION | 0.62 | 1.82 | 1.87E-02 |
| GOBP_MITOTIC_SPINDLE_ORGANIZATION | 0.62 | 1.80 | 1.87E-02 |
| GOBP_MONONUCLEAR_CELL_DIFFERENTIATION | 0.46 | 1.40 | 1.87E-02 |
| GOBP_MONONUCLEAR_CELL_MIGRATION | 0.47 | 1.39 | 1.87E-02 |
| GOBP_MRNA_EXPORT_FROM_NUCLEUS | 0.53 | 1.55 | 1.87E-02 |
| GOBP_MRNA_PROCESSING | 0.47 | 1.44 | 1.87E-02 |
| GOBP_MRNA_TRANSPORT | 0.52 | 1.52 | 1.87E-02 |
| GOBP_MYELOID_CELL_DIFFERENTIATION | 0.44 | 1.35 | 1.87E-02 |
| GOBP_MYELOID_LEUKOCYTE_DIFFERENTIATION | 0.47 | 1.40 | 1.87E-02 |
| GOBP_MYELOID_LEUKOCYTE_MIGRATION | 0.48 | 1.44 | 1.87E-02 |
| GOBP_NCRNA_METABOLIC_PROCESS | 0.44 | 1.34 | 1.87E-02 |
| GOBP_NCRNA_PROCESSING | 0.45 | 1.35 | 1.87E-02 |
| GOBP_NEGATIVE_REGULATION_OF_CELL_ACTIVATION | 0.49 | 1.47 | 1.87E-02 |
| GOBP_NEGATIVE_REGULATION_OF_CELL_ADHESION | 0.50 | 1.51 | 1.87E-02 |
| GOBP_NEGATIVE_REGULATION_OF_CELL_CELL_ADHESION | 0.50 | 1.47 | 1.87E-02 |
| GOBP_NEGATIVE_REGULATION_OF_CELL_CYCLE_G1_S_PHASE_TRANSITION | 0.56 | 1.64 | 1.87E-02 |
| GOBP_NEGATIVE_REGULATION_OF_CELL_CYCLE_PHASE_TRANSITION | 0.52 | 1.55 | 1.87E-02 |
| GOBP_NEGATIVE_REGULATION_OF_CELL_CYCLE_PROCESS | 0.50 | 1.53 | 1.87E-02 |
| GOBP_NEGATIVE_REGULATION_OF_CHROMOSOME_ORGANIZATION | 0.61 | 1.73 | 1.87E-02 |
| GOBP_NEGATIVE_REGULATION_OF_CYSTEINE_TYPE_ENDOPEPTIDASE_ACTIVITY | 0.54 | 1.55 | 1.87E-02 |
| GOBP_NEGATIVE_REGULATION_OF_IMMUNE_SYSTEM_PROCESS | 0.44 | 1.32 | 1.87E-02 |
| GOBP_NEGATIVE_REGULATION_OF_LEUKOCYTE_CELL_CELL_ADHESION | 0.50 | 1.46 | 1.87E-02 |
| GOBP_NEGATIVE_REGULATION_OF_LEUKOCYTE_PROLIFERATION | 0.56 | 1.61 | 1.87E-02 |
| GOBP_NEGATIVE_REGULATION_OF_LYMPHOCYTE_ACTIVATION | 0.51 | 1.49 | 1.87E-02 |
| GOBP_NEGATIVE_REGULATION_OF_MEIOTIC_CELL_CYCLE | 0.72 | 1.69 | 1.87E-02 |
| GOBP_NEGATIVE_REGULATION_OF_METAPHASE_ANAPHASE_TRANSITION_OF_CELL_CYCLE | 0.69 | 1.86 | 1.87E-02 |
| GOBP_NEGATIVE_REGULATION_OF_MITOTIC_CELL_CYCLE | 0.50 | 1.52 | 1.87E-02 |
| GOBP_NEGATIVE_REGULATION_OF_NUCLEAR_DIVISION | 0.69 | 1.91 | 1.87E-02 |
| GOBP_NEGATIVE_REGULATION_OF_ORGANELLE_ORGANIZATION | 0.46 | 1.40 | 1.87E-02 |
| GOBP_NEGATIVE_REGULATION_OF_SPROUTING_ANGIOGENESIS | 0.78 | 1.87 | 1.87E-02 |
| GOBP_NEURAL_TUBE_DEVELOPMENT | 0.47 | 1.39 | 1.87E-02 |
| GOBP_NEURAL_TUBE_FORMATION | 0.53 | 1.53 | 1.87E-02 |
| GOBP_NEUTRAL_AMINO_ACID_TRANSPORT | 0.61 | 1.65 | 1.87E-02 |
| GOBP_NEUTROPHIL_CHEMOTAXIS | 0.50 | 1.46 | 1.87E-02 |
| GOBP_NEUTROPHIL_MIGRATION | 0.49 | 1.43 | 1.87E-02 |
| GOBP_NUCLEAR_CHROMOSOME_SEGREGATION | 0.58 | 1.74 | 1.87E-02 |
| GOBP_NUCLEAR_EXPORT | 0.49 | 1.46 | 1.87E-02 |
| GOBP_NUCLEAR_TRANSPORT | 0.47 | 1.42 | 1.87E-02 |
| GOBP_NUCLEIC_ACID_PHOSPHODIESTER_BOND_HYDROLYSIS | 0.43 | 1.29 | 1.87E-02 |
| GOBP_NUCLEOBASE_CONTAINING_COMPOUND_TRANSPORT | 0.46 | 1.37 | 1.87E-02 |
| GOBP_NUCLEOSOME_ASSEMBLY | 0.66 | 1.84 | 1.87E-02 |
| GOBP_NUCLEOSOME_ORGANIZATION | 0.58 | 1.69 | 1.87E-02 |
| GOBP_ORGANELLE_FISSION | 0.53 | 1.62 | 1.87E-02 |
| GOBP_PEPTIDYL_LYSINE_MODIFICATION | 0.46 | 1.38 | 1.87E-02 |
| GOBP_PEPTIDYL_SERINE_MODIFICATION | 0.43 | 1.30 | 1.87E-02 |
| GOBP_PEPTIDYL_TYROSINE_MODIFICATION | 0.42 | 1.29 | 1.87E-02 |
| GOBP_PHAGOCYTOSIS | 0.46 | 1.38 | 1.87E-02 |
| GOBP_POSITIVE_REGULATION_OF_ADAPTIVE_IMMUNE_RESPONSE | 0.49 | 1.43 | 1.87E-02 |
| GOBP_POSITIVE_REGULATION_OF_B_CELL_ACTIVATION | 0.54 | 1.53 | 1.87E-02 |
| GOBP_POSITIVE_REGULATION_OF_CELL_ACTIVATION | 0.50 | 1.50 | 1.87E-02 |
| GOBP_POSITIVE_REGULATION_OF_CELL_ADHESION | 0.45 | 1.36 | 1.87E-02 |
| GOBP_POSITIVE_REGULATION_OF_CELL_CELL_ADHESION | 0.47 | 1.43 | 1.87E-02 |
| GOBP_POSITIVE_REGULATION_OF_CELL_CYCLE | 0.50 | 1.50 | 1.87E-02 |
| GOBP_POSITIVE_REGULATION_OF_CELL_CYCLE_ARREST | 0.57 | 1.64 | 1.87E-02 |
| GOBP_POSITIVE_REGULATION_OF_CELL_CYCLE_G2_M_PHASE_TRANSITION | 0.66 | 1.71 | 1.87E-02 |
| GOBP_POSITIVE_REGULATION_OF_CELL_CYCLE_PHASE_TRANSITION | 0.55 | 1.61 | 1.87E-02 |
| GOBP_POSITIVE_REGULATION_OF_CELL_CYCLE_PROCESS | 0.54 | 1.62 | 1.87E-02 |
| GOBP_POSITIVE_REGULATION_OF_CELL_PROJECTION_ORGANIZATION | 0.43 | 1.31 | 1.87E-02 |
| GOBP_POSITIVE_REGULATION_OF_CELLULAR_COMPONENT_BIOGENESIS | 0.42 | 1.28 | 1.87E-02 |
| GOBP_POSITIVE_REGULATION_OF_CELLULAR_PROTEIN_LOCALIZATION | 0.46 | 1.38 | 1.87E-02 |
| GOBP_POSITIVE_REGULATION_OF_CHEMOTAXIS | 0.50 | 1.46 | 1.87E-02 |
| GOBP_POSITIVE_REGULATION_OF_CHROMOSOME_ORGANIZATION | 0.48 | 1.43 | 1.87E-02 |
| GOBP_POSITIVE_REGULATION_OF_CYTOKINE_PRODUCTION | 0.45 | 1.37 | 1.87E-02 |
| GOBP_POSITIVE_REGULATION_OF_CYTOSKELETON_ORGANIZATION | 0.44 | 1.31 | 1.87E-02 |
| GOBP_POSITIVE_REGULATION_OF_DEFENSE_RESPONSE | 0.44 | 1.32 | 1.87E-02 |
| GOBP_POSITIVE_REGULATION_OF_DNA_METABOLIC_PROCESS | 0.49 | 1.47 | 1.87E-02 |
| GOBP_POSITIVE_REGULATION_OF_ERBB_SIGNALING_PATHWAY | 0.64 | 1.69 | 1.87E-02 |
| GOBP_POSITIVE_REGULATION_OF_GTPASE_ACTIVITY | 0.43 | 1.31 | 1.87E-02 |
| GOBP_POSITIVE_REGULATION_OF_HEMOPOIESIS | 0.52 | 1.53 | 1.87E-02 |
| GOBP_POSITIVE_REGULATION_OF_I_KAPPAB_KINASE_NF_KAPPAB_SIGNALING | 0.45 | 1.35 | 1.87E-02 |
| GOBP_POSITIVE_REGULATION_OF_IMMUNE_EFFECTOR_PROCESS | 0.45 | 1.35 | 1.87E-02 |
| GOBP_POSITIVE_REGULATION_OF_LEUKOCYTE_CELL_CELL_ADHESION | 0.50 | 1.49 | 1.87E-02 |
| GOBP_POSITIVE_REGULATION_OF_LEUKOCYTE_CHEMOTAXIS | 0.53 | 1.53 | 1.87E-02 |
| GOBP_POSITIVE_REGULATION_OF_LEUKOCYTE_MIGRATION | 0.51 | 1.50 | 1.87E-02 |
| GOBP_POSITIVE_REGULATION_OF_LEUKOCYTE_PROLIFERATION | 0.52 | 1.54 | 1.87E-02 |
| GOBP_POSITIVE_REGULATION_OF_LYMPHOCYTE_DIFFERENTIATION | 0.53 | 1.53 | 1.87E-02 |
| GOBP_POSITIVE_REGULATION_OF_MITOTIC_CELL_CYCLE | 0.52 | 1.53 | 1.87E-02 |
| GOBP_POSITIVE_REGULATION_OF_MYELOID_CELL_DIFFERENTIATION | 0.49 | 1.42 | 1.87E-02 |
| GOBP_POSITIVE_REGULATION_OF_PEPTIDYL_TYROSINE_PHOSPHORYLATION | 0.45 | 1.34 | 1.87E-02 |
| GOBP_POSITIVE_REGULATION_OF_PROTEIN_CONTAINING_COMPLEX_ASSEMBLY | 0.44 | 1.32 | 1.87E-02 |
| GOBP_POSITIVE_REGULATION_OF_PROTEIN_KINASE_ACTIVITY | 0.42 | 1.29 | 1.87E-02 |
| GOBP_POSITIVE_REGULATION_OF_PROTEIN_LOCALIZATION_TO_NUCLEUS | 0.51 | 1.45 | 1.87E-02 |
| GOBP_POSITIVE_REGULATION_OF_RESPONSE_TO_BIOTIC_STIMULUS | 0.44 | 1.33 | 1.87E-02 |
| GOBP_POSITIVE_REGULATION_OF_RESPONSE_TO_EXTERNAL_STIMULUS | 0.43 | 1.31 | 1.87E-02 |
| GOBP_POSITIVE_REGULATION_OF_T_CELL_PROLIFERATION | 0.55 | 1.58 | 1.87E-02 |
| GOBP_PRIMARY_NEURAL_TUBE_FORMATION | 0.53 | 1.53 | 1.87E-02 |
| GOBP_PRODUCTION_OF_MOLECULAR_MEDIATOR_OF_IMMUNE_RESPONSE | 0.46 | 1.36 | 1.87E-02 |
| GOBP_PROSTAGLANDIN_SECRETION | 0.78 | 1.77 | 1.87E-02 |
| GOBP_PROSTAGLANDIN_TRANSPORT | 0.73 | 1.72 | 1.87E-02 |
| GOBP_PROTEIN_ACETYLATION | 0.47 | 1.39 | 1.87E-02 |
| GOBP_PROTEIN_ACYLATION | 0.44 | 1.32 | 1.87E-02 |
| GOBP_PROTEIN_CONTAINING_COMPLEX_LOCALIZATION | 0.48 | 1.45 | 1.87E-02 |
| GOBP_PROTEIN_DNA_COMPLEX_SUBUNIT_ORGANIZATION | 0.52 | 1.56 | 1.87E-02 |
| GOBP_PROTEIN_LOCALIZATION_TO_CHROMOSOME | 0.53 | 1.52 | 1.87E-02 |
| GOBP_PROTEIN_LOCALIZATION_TO_CHROMOSOME_CENTROMERIC_REGION | 0.71 | 1.69 | 1.87E-02 |
| GOBP_PROTEIN_LOCALIZATION_TO_NUCLEUS | 0.45 | 1.36 | 1.87E-02 |
| GOBP_PROTEIN_POLYMERIZATION | 0.44 | 1.33 | 1.87E-02 |
| GOBP_RAS_PROTEIN_SIGNAL_TRANSDUCTION | 0.43 | 1.31 | 1.87E-02 |
| GOBP_RECOMBINATIONAL_REPAIR | 0.54 | 1.58 | 1.87E-02 |
| GOBP_REGULATION_OF_ACTIN_FILAMENT_BASED_PROCESS | 0.42 | 1.27 | 1.87E-02 |
| GOBP_REGULATION_OF_ACTIN_FILAMENT_LENGTH | 0.46 | 1.37 | 1.87E-02 |
| GOBP_REGULATION_OF_ACTIN_FILAMENT_ORGANIZATION | 0.43 | 1.30 | 1.87E-02 |
| GOBP_REGULATION_OF_ALPHA_BETA_T_CELL_ACTIVATION | 0.50 | 1.46 | 1.87E-02 |
| GOBP_REGULATION_OF_ATTACHMENT_OF_SPINDLE_MICROTUBULES_TO_KINETOCHORE | 0.78 | 1.72 | 1.88E-02 |
| GOBP_REGULATION_OF_B_CELL_ACTIVATION | 0.54 | 1.58 | 1.87E-02 |
| GOBP_REGULATION_OF_CELL_CELL_ADHESION | 0.47 | 1.44 | 1.87E-02 |
| GOBP_REGULATION_OF_CELL_CYCLE_ARREST | 0.54 | 1.57 | 1.87E-02 |
| GOBP_REGULATION_OF_CELL_CYCLE_G1_S_PHASE_TRANSITION | 0.50 | 1.49 | 1.87E-02 |
| GOBP_REGULATION_OF_CELL_CYCLE_G2_M_PHASE_TRANSITION | 0.50 | 1.49 | 1.87E-02 |
| GOBP_REGULATION_OF_CELL_CYCLE_PHASE_TRANSITION | 0.51 | 1.55 | 1.87E-02 |
| GOBP_REGULATION_OF_CELL_DEVELOPMENT | 0.40 | 1.22 | 1.87E-02 |
| GOBP_REGULATION_OF_CELL_DIVISION | 0.49 | 1.47 | 1.87E-02 |
| GOBP_REGULATION_OF_CELL_MORPHOGENESIS | 0.43 | 1.30 | 1.87E-02 |
| GOBP_REGULATION_OF_CELL_SUBSTRATE_ADHESION | 0.46 | 1.37 | 1.87E-02 |
| GOBP_REGULATION_OF_CELLULAR_COMPONENT_SIZE | 0.44 | 1.33 | 1.87E-02 |
| GOBP_REGULATION_OF_CELLULAR_PROTEIN_LOCALIZATION | 0.41 | 1.25 | 1.87E-02 |
| GOBP_REGULATION_OF_CELLULAR_RESPONSE_TO_HEAT | 0.52 | 1.49 | 1.87E-02 |
| GOBP_REGULATION_OF_CHEMOTAXIS | 0.47 | 1.40 | 1.87E-02 |
| GOBP_REGULATION_OF_CHROMOSOME_ORGANIZATION | 0.52 | 1.57 | 1.87E-02 |
| GOBP_REGULATION_OF_CHROMOSOME_SEGREGATION | 0.62 | 1.79 | 1.87E-02 |
| GOBP_REGULATION_OF_CHROMOSOME_SEPARATION | 0.63 | 1.78 | 1.87E-02 |
| GOBP_REGULATION_OF_CYCLIN_DEPENDENT_PROTEIN_KINASE_ACTIVITY | 0.58 | 1.69 | 1.87E-02 |
| GOBP_REGULATION_OF_CYSTEINE_TYPE_ENDOPEPTIDASE_ACTIVITY | 0.47 | 1.39 | 1.87E-02 |
| GOBP_REGULATION_OF_CYTOKINESIS | 0.52 | 1.51 | 1.87E-02 |
| GOBP_REGULATION_OF_CYTOSKELETON_ORGANIZATION | 0.45 | 1.37 | 1.87E-02 |
| GOBP_REGULATION_OF_DNA_BINDING_TRANSCRIPTION_FACTOR_ACTIVITY | 0.42 | 1.28 | 1.87E-02 |
| GOBP_REGULATION_OF_DNA_BIOSYNTHETIC_PROCESS | 0.50 | 1.44 | 1.87E-02 |
| GOBP_REGULATION_OF_DNA_DEPENDENT_DNA_REPLICATION | 0.62 | 1.70 | 1.87E-02 |
| GOBP_REGULATION_OF_DNA_METABOLIC_PROCESS | 0.48 | 1.45 | 1.87E-02 |
| GOBP_REGULATION_OF_DNA_RECOMBINATION | 0.51 | 1.45 | 1.87E-02 |
| GOBP_REGULATION_OF_DNA_REPAIR | 0.50 | 1.46 | 1.87E-02 |
| GOBP_REGULATION_OF_DNA_REPLICATION | 0.53 | 1.55 | 1.87E-02 |
| GOBP_REGULATION_OF_ERBB_SIGNALING_PATHWAY | 0.51 | 1.48 | 1.87E-02 |
| GOBP_REGULATION_OF_GENE_EXPRESSION_EPIGENETIC | 0.48 | 1.40 | 1.87E-02 |
| GOBP_REGULATION_OF_GRANULOCYTE_DIFFERENTIATION | 0.74 | 1.69 | 1.87E-02 |
| GOBP_REGULATION_OF_GTPASE_ACTIVITY | 0.44 | 1.33 | 1.87E-02 |
| GOBP_REGULATION_OF_HEMOPOIESIS | 0.45 | 1.38 | 1.87E-02 |
| GOBP_REGULATION_OF_INNATE_IMMUNE_RESPONSE | 0.45 | 1.36 | 1.87E-02 |
| GOBP_REGULATION_OF_LEUKOCYTE_APOPTOTIC_PROCESS | 0.51 | 1.47 | 1.87E-02 |
| GOBP_REGULATION_OF_LEUKOCYTE_DIFFERENTIATION | 0.48 | 1.46 | 1.87E-02 |
| GOBP_REGULATION_OF_LEUKOCYTE_MIGRATION | 0.50 | 1.48 | 1.87E-02 |
| GOBP_REGULATION_OF_LEUKOCYTE_PROLIFERATION | 0.50 | 1.51 | 1.87E-02 |
| GOBP_REGULATION_OF_LYMPHOCYTE_ACTIVATION | 0.48 | 1.47 | 1.87E-02 |
| GOBP_REGULATION_OF_LYMPHOCYTE_DIFFERENTIATION | 0.50 | 1.47 | 1.87E-02 |
| GOBP_REGULATION_OF_MEIOTIC_CELL_CYCLE | 0.64 | 1.73 | 1.87E-02 |
| GOBP_REGULATION_OF_MEIOTIC_NUCLEAR_DIVISION | 0.65 | 1.67 | 1.87E-02 |
| GOBP_REGULATION_OF_MICROTUBULE_CYTOSKELETON_ORGANIZATION | 0.48 | 1.41 | 1.87E-02 |
| GOBP_REGULATION_OF_MITOTIC_NUCLEAR_DIVISION | 0.61 | 1.78 | 1.87E-02 |
| GOBP_REGULATION_OF_MITOTIC_SISTER_CHROMATID_SEGREGATION | 0.68 | 1.86 | 1.87E-02 |
| GOBP_REGULATION_OF_MONONUCLEAR_CELL_MIGRATION | 0.54 | 1.58 | 1.87E-02 |
| GOBP_REGULATION_OF_MRNA_METABOLIC_PROCESS | 0.43 | 1.31 | 1.87E-02 |
| GOBP_REGULATION_OF_MYELOID_CELL_DIFFERENTIATION | 0.44 | 1.31 | 1.87E-02 |
| GOBP_REGULATION_OF_MYELOID_LEUKOCYTE_DIFFERENTIATION | 0.50 | 1.46 | 1.87E-02 |
| GOBP_REGULATION_OF_NEURON_PROJECTION_DEVELOPMENT | 0.42 | 1.27 | 1.87E-02 |
| GOBP_REGULATION_OF_NUCLEAR_DIVISION | 0.59 | 1.74 | 1.87E-02 |
| GOBP_REGULATION_OF_ORGANELLE_ASSEMBLY | 0.46 | 1.35 | 1.87E-02 |
| GOBP_REGULATION_OF_PEPTIDYL_TYROSINE_PHOSPHORYLATION | 0.43 | 1.29 | 1.87E-02 |
| GOBP_REGULATION_OF_PHAGOCYTOSIS | 0.52 | 1.51 | 1.87E-02 |
| GOBP_REGULATION_OF_PROSTAGLANDIN_SECRETION | 0.78 | 1.69 | 1.88E-02 |
| GOBP_REGULATION_OF_PROTEIN_CONTAINING_COMPLEX_ASSEMBLY | 0.43 | 1.30 | 1.87E-02 |
| GOBP_REGULATION_OF_PROTEIN_LOCALIZATION_TO_NUCLEUS | 0.49 | 1.44 | 1.87E-02 |
| GOBP_REGULATION_OF_PROTEIN_POLYMERIZATION | 0.45 | 1.35 | 1.87E-02 |
| GOBP_REGULATION_OF_PROTEIN_SERINE_THREONINE_KINASE_ACTIVITY | 0.42 | 1.29 | 1.87E-02 |
| GOBP_REGULATION_OF_RESPONSE_TO_BIOTIC_STIMULUS | 0.42 | 1.28 | 1.87E-02 |
| GOBP_REGULATION_OF_RESPONSE_TO_CYTOKINE_STIMULUS | 0.50 | 1.48 | 1.87E-02 |
| GOBP_REGULATION_OF_RESPONSE_TO_DNA_DAMAGE_STIMULUS | 0.48 | 1.42 | 1.87E-02 |
| GOBP_REGULATION_OF_SIGNAL_TRANSDUCTION_BY_P53_CLASS_MEDIATOR | 0.51 | 1.50 | 1.87E-02 |
| GOBP_REGULATION_OF_SMALL_GTPASE_MEDIATED_SIGNAL_TRANSDUCTION | 0.44 | 1.34 | 1.87E-02 |
| GOBP_REGULATION_OF_SUPRAMOLECULAR_FIBER_ORGANIZATION | 0.43 | 1.29 | 1.87E-02 |
| GOBP_REGULATION_OF_T_CELL_ACTIVATION | 0.48 | 1.46 | 1.87E-02 |
| GOBP_REGULATION_OF_T_CELL_DIFFERENTIATION | 0.50 | 1.48 | 1.87E-02 |
| GOBP_REGULATION_OF_TRANSCRIPTION_INVOLVED_IN_G1_S_TRANSITION_OF_MITOTIC_CELL_CYCLE | 0.68 | 1.74 | 1.87E-02 |
| GOBP_REGULATORY_T_CELL_DIFFERENTIATION | 0.66 | 1.73 | 1.87E-02 |
| GOBP_RESPIRATORY_BURST | 0.70 | 1.82 | 1.87E-02 |
| GOBP_RESPONSE_TO_LEUKEMIA_INHIBITORY_FACTOR | 0.52 | 1.48 | 1.87E-02 |
| GOBP_RIBONUCLEOPROTEIN_COMPLEX_BIOGENESIS | 0.45 | 1.38 | 1.87E-02 |
| GOBP_RIBONUCLEOPROTEIN_COMPLEX_SUBUNIT_ORGANIZATION | 0.46 | 1.35 | 1.87E-02 |
| GOBP_RIBOSOME_BIOGENESIS | 0.44 | 1.31 | 1.87E-02 |
| GOBP_RNA_3_END_PROCESSING | 0.50 | 1.45 | 1.87E-02 |
| GOBP_RNA_EXPORT_FROM_NUCLEUS | 0.54 | 1.56 | 1.87E-02 |
| GOBP_RNA_LOCALIZATION | 0.52 | 1.56 | 1.87E-02 |
| GOBP_RNA_SPLICING | 0.47 | 1.44 | 1.87E-02 |
| GOBP_RNA_SPLICING_VIA_TRANSESTERIFICATION_REACTIONS | 0.49 | 1.47 | 1.87E-02 |
| GOBP_RRNA_METABOLIC_PROCESS | 0.45 | 1.33 | 1.87E-02 |
| GOBP_SERINE_TRANSPORT | 0.80 | 1.70 | 1.88E-02 |
| GOBP_SIGNAL_TRANSDUCTION_BY_P53_CLASS_MEDIATOR | 0.50 | 1.49 | 1.87E-02 |
| GOBP_SIGNAL_TRANSDUCTION_IN_RESPONSE_TO_DNA_DAMAGE | 0.52 | 1.52 | 1.87E-02 |
| GOBP_SIGNAL_TRANSDUCTION_INVOLVED_IN_CELL_CYCLE_CHECKPOINT | 0.59 | 1.66 | 1.87E-02 |
| GOBP_SISTER_CHROMATID_SEGREGATION | 0.58 | 1.72 | 1.87E-02 |
| GOBP_SMALL_GTPASE_MEDIATED_SIGNAL_TRANSDUCTION | 0.44 | 1.35 | 1.87E-02 |
| GOBP_SPINDLE_ASSEMBLY | 0.54 | 1.57 | 1.87E-02 |
| GOBP_SPINDLE_MIDZONE_ASSEMBLY | 0.83 | 1.79 | 1.88E-02 |
| GOBP_SPINDLE_ORGANIZATION | 0.57 | 1.68 | 1.87E-02 |
| GOBP_SUPEROXIDE_ANION_GENERATION | 0.68 | 1.77 | 1.87E-02 |
| GOBP_SUPEROXIDE_METABOLIC_PROCESS | 0.62 | 1.75 | 1.87E-02 |
| GOBP_T_CELL_ACTIVATION | 0.48 | 1.46 | 1.87E-02 |
| GOBP_T_CELL_CHEMOTAXIS | 0.65 | 1.66 | 1.87E-02 |
| GOBP_T_CELL_DIFFERENTIATION | 0.48 | 1.44 | 1.87E-02 |
| GOBP_T_CELL_PROLIFERATION | 0.49 | 1.44 | 1.87E-02 |
| GOBP_T_CELL_RECEPTOR_SIGNALING_PATHWAY | 0.46 | 1.36 | 1.87E-02 |
| GOBP_TELOMERE_ORGANIZATION | 0.55 | 1.63 | 1.87E-02 |
| GOCC_ACTIN_CYTOSKELETON | 0.40 | 1.22 | 1.87E-02 |
| GOCC_ACTIN_FILAMENT | 0.53 | 1.55 | 1.87E-02 |
| GOCC_CATALYTIC_STEP_2_SPLICEOSOME | 0.53 | 1.51 | 1.87E-02 |
| GOCC_CELL_LEADING_EDGE | 0.46 | 1.39 | 1.87E-02 |
| GOCC_CELL_SUBSTRATE_JUNCTION | 0.42 | 1.26 | 1.87E-02 |
| GOCC_CENTRIOLAR_SATELLITE | 0.49 | 1.44 | 1.87E-02 |
| GOCC_CENTRIOLE | 0.50 | 1.45 | 1.87E-02 |
| GOCC_CHROMOCENTER | 0.78 | 1.81 | 1.87E-02 |
| GOCC_CHROMOSOMAL_REGION | 0.57 | 1.72 | 1.87E-02 |
| GOCC_CHROMOSOME_CENTROMERIC_REGION | 0.62 | 1.82 | 1.87E-02 |
| GOCC_CHROMOSOME_TELOMERIC_REGION | 0.52 | 1.52 | 1.87E-02 |
| GOCC_CMG_COMPLEX | 0.93 | 1.98 | 1.88E-02 |
| GOCC_COLLAGEN_TRIMER | 0.53 | 1.52 | 1.87E-02 |
| GOCC_COMPLEX_OF_COLLAGEN_TRIMERS | 0.69 | 1.69 | 1.87E-02 |
| GOCC_CONDENSED_CHROMOSOME | 0.59 | 1.76 | 1.87E-02 |
| GOCC_CONDENSED_CHROMOSOME_CENTROMERIC_REGION | 0.70 | 2.01 | 1.87E-02 |
| GOCC_CONDENSED_NUCLEAR_CHROMOSOME | 0.58 | 1.66 | 1.87E-02 |
| GOCC_CONDENSED_NUCLEAR_CHROMOSOME_KINETOCHORE | 0.82 | 1.90 | 1.87E-02 |
| GOCC_DNA_PACKAGING_COMPLEX | 0.72 | 1.75 | 1.87E-02 |
| GOCC_DNA_REPLICATION_PREINITIATION_COMPLEX | 0.93 | 1.98 | 1.88E-02 |
| GOCC_FIBRILLAR_COLLAGEN_TRIMER | 0.84 | 1.84 | 1.88E-02 |
| GOCC_HETEROCHROMATIN | 0.58 | 1.63 | 1.87E-02 |
| GOCC_KINESIN_COMPLEX | 0.60 | 1.64 | 1.87E-02 |
| GOCC_KINETOCHORE | 0.65 | 1.89 | 1.87E-02 |
| GOCC_LAMELLIPODIUM | 0.49 | 1.46 | 1.87E-02 |
| GOCC_MHC_CLASS_II_PROTEIN_COMPLEX | 0.80 | 1.81 | 1.87E-02 |
| GOCC_MHC_PROTEIN_COMPLEX | 0.70 | 1.74 | 1.87E-02 |
| GOCC_MICROTUBULE | 0.47 | 1.43 | 1.87E-02 |
| GOCC_MICROTUBULE_ASSOCIATED_COMPLEX | 0.49 | 1.43 | 1.87E-02 |
| GOCC_MICROVILLUS_MEMBRANE | 0.68 | 1.71 | 1.87E-02 |
| GOCC_MIDBODY | 0.47 | 1.38 | 1.87E-02 |
| GOCC_MITOTIC_SPINDLE | 0.58 | 1.71 | 1.87E-02 |
| GOCC_MITOTIC_SPINDLE_MIDZONE | 0.80 | 1.78 | 1.87E-02 |
| GOCC_NUCLEAR_CHROMOSOME | 0.56 | 1.66 | 1.87E-02 |
| GOCC_NUCLEAR_ENVELOPE | 0.41 | 1.25 | 1.87E-02 |
| GOCC_NUCLEAR_PERIPHERY | 0.54 | 1.56 | 1.87E-02 |
| GOCC_NUCLEAR_SPECK | 0.43 | 1.30 | 1.87E-02 |
| GOCC_PHAGOCYTIC_VESICLE | 0.47 | 1.39 | 1.87E-02 |
| GOCC_REPLICATION_FORK | 0.62 | 1.72 | 1.87E-02 |
| GOCC_RNA_POLYMERASE_II_TRANSCRIPTION_REGULATOR_COMPLEX | 0.47 | 1.40 | 1.87E-02 |
| GOCC_RUFFLE | 0.49 | 1.46 | 1.87E-02 |
| GOCC_SPECIFIC_GRANULE_MEMBRANE | 0.51 | 1.47 | 1.87E-02 |
| GOCC_SPINDLE | 0.53 | 1.59 | 1.87E-02 |
| GOCC_SPINDLE_MICROTUBULE | 0.61 | 1.69 | 1.87E-02 |
| GOCC_SPINDLE_MIDZONE | 0.72 | 1.89 | 1.87E-02 |
| GOCC_SPINDLE_POLE | 0.54 | 1.60 | 1.87E-02 |
| GOCC_SPLICEOSOMAL_COMPLEX | 0.50 | 1.49 | 1.87E-02 |
| GOCC_TERTIARY_GRANULE | 0.49 | 1.45 | 1.87E-02 |
| GOCC_TRANSCRIPTION_REGULATOR_COMPLEX | 0.44 | 1.35 | 1.87E-02 |
| GOCC_U2_TYPE_SPLICEOSOMAL_COMPLEX | 0.50 | 1.45 | 1.87E-02 |
| GOMF_ACTIN_BINDING | 0.42 | 1.29 | 1.87E-02 |
| GOMF_CADHERIN_BINDING | 0.45 | 1.37 | 1.87E-02 |
| GOMF_CATALYTIC_ACTIVITY_ACTING_ON_DNA | 0.48 | 1.44 | 1.87E-02 |
| GOMF_CATALYTIC_ACTIVITY_ACTING_ON_RNA | 0.43 | 1.29 | 1.87E-02 |
| GOMF_CELL_ADHESION_MOLECULE_BINDING | 0.44 | 1.34 | 1.87E-02 |
| GOMF_DNA_DEPENDENT_ATPASE_ACTIVITY | 0.63 | 1.72 | 1.87E-02 |
| GOMF_DNA_HELICASE_ACTIVITY | 0.55 | 1.56 | 1.87E-02 |
| GOMF_DNA_REPLICATION_ORIGIN_BINDING | 0.83 | 1.92 | 1.87E-02 |
| GOMF_DNA_SECONDARY_STRUCTURE_BINDING | 0.64 | 1.66 | 1.87E-02 |
| GOMF_ENZYME_ACTIVATOR_ACTIVITY | 0.41 | 1.27 | 1.87E-02 |
| GOMF_EXTRACELLULAR_MATRIX_STRUCTURAL_CONSTITUENT | 0.51 | 1.50 | 1.87E-02 |
| GOMF_GTPASE_ACTIVATOR_ACTIVITY | 0.45 | 1.36 | 1.87E-02 |
| GOMF_HELICASE_ACTIVITY | 0.49 | 1.45 | 1.87E-02 |
| GOMF_HISTONE_BINDING | 0.51 | 1.51 | 1.87E-02 |
| GOMF_IMMUNE_RECEPTOR_ACTIVITY | 0.48 | 1.41 | 1.87E-02 |
| GOMF_MAGNESIUM_ION_BINDING | 0.44 | 1.32 | 1.87E-02 |
| GOMF_MICROTUBULE_BINDING | 0.49 | 1.46 | 1.87E-02 |
| GOMF_NEUTRAL_AMINO_ACID_TRANSMEMBRANE_TRANSPORTER_ACTIVITY | 0.67 | 1.76 | 1.87E-02 |
| GOMF_NUCLEOSIDE_TRIPHOSPHATASE_REGULATOR_ACTIVITY | 0.42 | 1.27 | 1.87E-02 |
| GOMF_PEPTIDE_ANTIGEN_BINDING | 0.70 | 1.76 | 1.87E-02 |
| GOMF_PROTEIN_SERINE_KINASE_ACTIVITY | 0.45 | 1.35 | 1.87E-02 |
| GOMF_PROTEIN_SERINE_THREONINE_KINASE_ACTIVITY | 0.43 | 1.32 | 1.87E-02 |
| GOMF_RAGE_RECEPTOR_BINDING | 0.83 | 1.77 | 1.88E-02 |
| GOMF_SH3_DOMAIN_BINDING | 0.51 | 1.50 | 1.87E-02 |
| GOMF_SINGLE_STRANDED_DNA_BINDING | 0.52 | 1.50 | 1.87E-02 |
| GOMF_SINGLE_STRANDED_DNA_HELICASE_ACTIVITY | 0.76 | 1.82 | 1.87E-02 |
| GOMF_TRANSCRIPTION_COACTIVATOR_ACTIVITY | 0.43 | 1.29 | 1.87E-02 |
| GOMF_TRANSCRIPTION_COREGULATOR_ACTIVITY | 0.42 | 1.28 | 1.87E-02 |
| GOMF_TUBULIN_BINDING | 0.48 | 1.46 | 1.87E-02 |
| KEGG_ALLOGRAFT_REJECTION | 0.59 | 1.57 | 4.55E-02 |
| KEGG_ANTIGEN_PROCESSING_AND_PRESENTATION | 0.52 | 1.49 | 1.90E-02 |
| KEGG_ASTHMA | 0.67 | 1.73 | 1.29E-02 |
| KEGG_AUTOIMMUNE_THYROID_DISEASE | 0.61 | 1.66 | 1.29E-02 |
| KEGG_CELL_ADHESION_MOLECULES_CAMS | 0.50 | 1.49 | 1.29E-02 |
| KEGG_CELL_CYCLE | 0.63 | 1.85 | 1.29E-02 |
| KEGG_CHEMOKINE_SIGNALING_PATHWAY | 0.46 | 1.38 | 1.29E-02 |
| KEGG_CYTOKINE_CYTOKINE_RECEPTOR_INTERACTION | 0.42 | 1.27 | 4.92E-02 |
| KEGG_DNA_REPLICATION | 0.68 | 1.80 | 1.29E-02 |
| KEGG_ECM_RECEPTOR_INTERACTION | 0.57 | 1.63 | 1.29E-02 |
| KEGG_FC_GAMMA_R_MEDIATED_PHAGOCYTOSIS | 0.52 | 1.50 | 1.29E-02 |
| KEGG_GRAFT_VERSUS_HOST_DISEASE | 0.62 | 1.66 | 1.29E-02 |
| KEGG_HEMATOPOIETIC_CELL_LINEAGE | 0.57 | 1.64 | 1.29E-02 |
| KEGG_INTESTINAL_IMMUNE_NETWORK_FOR_IGA_PRODUCTION | 0.61 | 1.67 | 1.90E-02 |
| KEGG_LEISHMANIA_INFECTION | 0.61 | 1.73 | 1.29E-02 |
| KEGG_LEUKOCYTE_TRANSENDOTHELIAL_MIGRATION | 0.47 | 1.39 | 3.13E-02 |
| KEGG_MISMATCH_REPAIR | 0.65 | 1.64 | 1.29E-02 |
| KEGG_PANCREATIC_CANCER | 0.51 | 1.45 | 2.51E-02 |
| KEGG_PATHWAYS_IN_CANCER | 0.43 | 1.30 | 1.90E-02 |
| KEGG_PRIMARY_IMMUNODEFICIENCY | 0.64 | 1.70 | 1.90E-02 |
| KEGG_PROGESTERONE_MEDIATED_OOCYTE_MATURATION | 0.51 | 1.48 | 1.29E-02 |
| KEGG_SMALL_CELL_LUNG_CANCER | 0.50 | 1.45 | 1.90E-02 |
| KEGG_SPLICEOSOME | 0.52 | 1.53 | 1.29E-02 |
| KEGG_T_CELL_RECEPTOR_SIGNALING_PATHWAY | 0.50 | 1.47 | 1.29E-02 |
| KEGG_TOLL_LIKE_RECEPTOR_SIGNALING_PATHWAY | 0.48 | 1.39 | 2.51E-02 |
| KEGG_TYPE_I_DIABETES_MELLITUS | 0.57 | 1.55 | 3.13E-02 |
